# Modeling and Optimization for a New Compliant 2-dof Stage for Locating Biomaterial Samples by an Efficient Approach of a Kinetostatic Analysis-Based Method and Neural Network Algorithm

**DOI:** 10.1155/2022/6709464

**Published:** 2022-08-16

**Authors:** Minh Phung Dang, Hieu Giang Le, Minh Nhut Van, Ngoc Le Chau, Thanh-Phong Dao

**Affiliations:** ^1^Faculty of Mechanical Engineering, Ho Chi Minh City University of Technology and Education, Ho Chi Minh City, Vietnam; ^2^Faculty of Mechanical Engineering, Industrial University of Ho Chi Minh City, Ho Chi Minh City, Vietnam; ^3^Division of Computational Mechatronics, Institute for Computational Science, Ton Duc Thang University, Ho Chi Minh City, Vietnam; ^4^Faculty of Electrical & Electronics Engineering, Ton Duc Thang University, Ho Chi Minh City, Vietnam

## Abstract

The nanoindentation technique is employed to characterize the behaviors of biomaterials. Nevertheless, there is a lack of development of a miniaturized precise positioner for in situ nanoindentation. Besides, modeling behaviors of the positioner are restricted due to its complex kinematic characteristics. Therefore, this paper proposes a novel compliant two degrees of freedom (dof) stage for positioning a biomaterial sample in in situ nanoindentation. In addition, a new modeling and dimensional optimization synthesis method of the stage is developed. The proposed effective methodology is developed based on a kinetostatic analysis-based calculation method, the Lagrange approach, and a neural network algorithm. With an increased advance in artificial intelligence, a neural network algorithm is proposed to extend the applicability of artificial neural networks in optimizing the parameters of the stage. First, the 2-dof stage is built via a combination of an eight-lever displacement amplifier and a symmetric parallelogram mechanism. Second, a chain of mathematical equations of the 2-dof stage is constructed using a kinetostatic analysis-based method to calculate the ratio of displacement amplification and the input stiffness of the 2-dof stage. Then, the Lagrange method is utilized to formulate the dynamic equation of the 2-dof stage. Finally, a neural network algorithm is adopted to maximize the natural first frequency of the proposed stage. The optimal results determined that the frequency of the stage can achieve a high value of 112.0995 Hz. Besides, the formed mathematical models were relatively precise by comprising the simulation verifications.

## 1. Introduction

Advances in in situ nanoindentation testers have resulted in great interest in developing compact-size mechanical mechanisms with better performances. The goals of a compact-size mechanism are to obtain lightweight, low cost, and to able be integrated into nanoindentation devices in observing the online mechanical behaviors of a material sample [1]. Especially, in situ nanoindentation checking plays a vital role in testing for behaviors of biomaterials in implants (e.g., bone, teeth, femur, prosthetics, and so forth) [2, 3]. In a general in situ nanoindentation system, there are two main mechanisms/stages, including the one degree of freedom (dof) fine stage for bringing the indenter and the 2-dof fine stage for locating the material sample [4].

With the aforementioned regards in mind, many researchers have made significant 2-dof mechanical stages in developing the in situ nanoindentation. Huang et al. [5] used the concept of flexure hinge-based compliant mechanisms to design a 1-dof stage for driving the indenter. Then, they continued to develop a new 1-dof stage with specifications of crystal silicon [6]. Moreover, Huang et al. [6] designed a 1-dof stage for characterizing behaviors of metallic glass. In these studies, they mainly employed the compliant mechanism in developing the 1-dof stage for utilizing the indenter driver. It is well known that compliant mechanisms offer nonassembled structure, zero friction, and simple manufacture which are advantages to overcome the limitations of rigid-counterparts in precise mechanical engineering. In the last decade, flexure hinge-based compliant mechanisms have been widely utilized for precise engineering and fine positioner with compact sizes such as a positioner [7, 8] and valve [6, 9].

At present, many different compliant 2-dof stages have been developed for micro/nanomanipulations. Similar to these applications, a piezoelectric actuator (PZT) is often chosen due to its high precision and fast response. Nevertheless, one of the drawbacks of the PZTs is a limited output stroke [10]. This causes a limitation of providing a large input displacement for the 2-dof stages. As a result, a few common amplifiers were developed, such as lever, bridge, and hybrid lever-bridge types [11]. Considering the design of compliant 2-dof stages, many research groups have focused on developing the series-parallel kinematic chains in coupling with the PZTs to gain the high performances. For instance, Ling et al. [12] developed a 2-dof stage with good specifications. They used the pseudo-rigid-body model and the Lagrange method to analyze the kinetostatic and dynamic behaviors of the stage. Qu et al. [13] designed a 2-dof stage using the parallelogram scheme. They analyzed the kinematics, stiffness, and workspace of the 2-dof stage through the matrix method. More recently, Wang et al. [14] designed a 2-dof stage, and the compliance matrix was employed for characterizing the stiffness. Xiao et al. [15] developed a 2-dof stage, and the stiffness as well as kinematic models was formulated through the matrix displacement method. Although the previous 2-dof stages were well developed, they were almost tended for developing the micro/nanomanipulations. Based on the aforementioned studies, it can be seen that there is a lack of deep investigations in developing compliant 2-dof stages for use in locating biomaterials in the in situ nanoindentation tester industry.

This paper aimed to develop a new compliant 2-dof stage which can be employed in locating biomaterials in in situ nanoindentation systems. The design details of the stage are developed based on the multiple lever amplification mechanism and series-parallel chain. The kinetostatics and dynamics are derived based on the deformable mechanics, the elastic beam theory, and the Lagrange approach.

The main contributions of this paper are summarized as follows: (i) A new compliant 02-dof stage with great dynamic characteristics is designed for locating biospecimens in nanoindentation tester systems. (ii) The stiffness and amplification ratio are established. Then, the dynamic equation of the 2-dof stage is formulated. The correctness of the established mathematical models are verified through finite element simulations. (iii) Finally, the main parameters of the 2-dof stage are optimized using the neural network algorithm which benefits excellent behaviors of an artificial neural network.

## 2. Conceptual Design of a Compliant 2-dof Stage

### 2.1. Design Scheme of the 1-dof Mechanism

**Figure 1:**
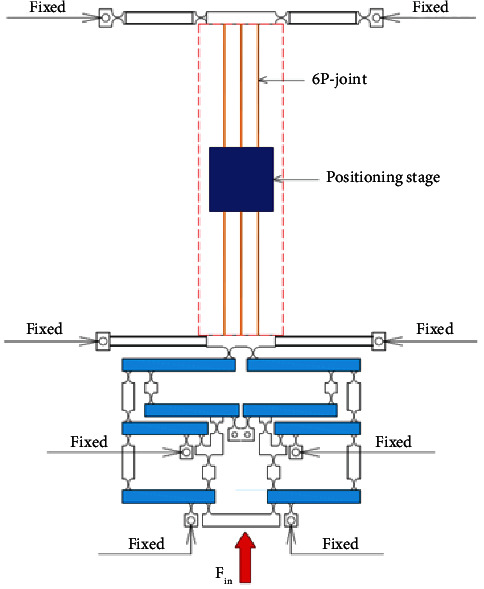
Design scheme of the 1-dof symmetrical mechanism.

Figure 1 describes the operating mechanism of a 1-dof stage which comprises an eight-lever displacement amplifier and a parallelogram mechanism. The amplifier is aimed to amplify the displacement, so-called stroke, of the proposed 1-dof stage. Meanwhile, the parallelogram mechanism is employed to generate good translation in the desirable motion and reduce the undesirable motions. It means that this design scheme ensures a good design with a large amplification ratio as well as reduces the parasitic motion error.

### 2.2. Operation Scheme of the 2-dof Stage

**Figure 2:**
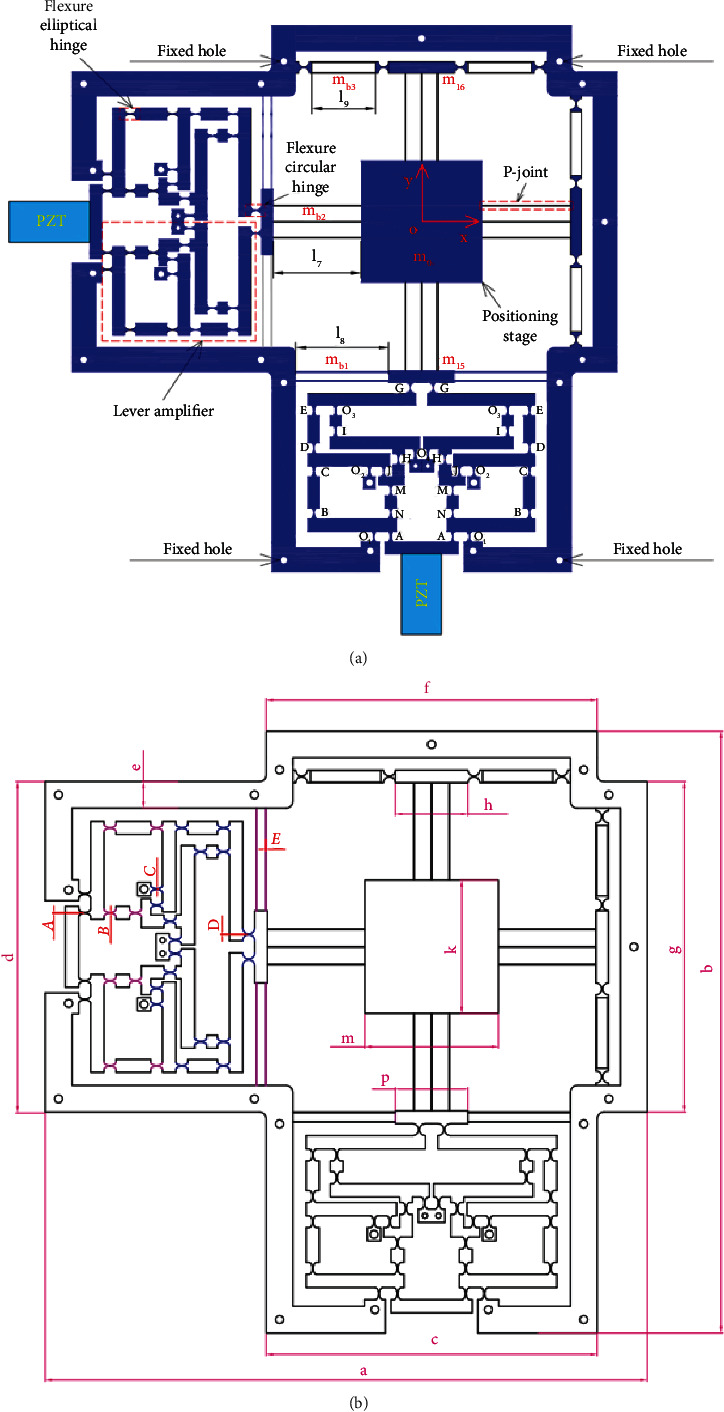
The proposed 2-dof stage: (a) design diagram and (b) main dimensioning parameters.

Based on the design and operation schemes of the 1-dof stage in Figure 1, the operation principle and design scheme of a new 2-dof stage are provided in Figure 2(a). The design of the 2-dof stage is a module-based scheme. The module of the 1-dof stage is symmetrically arranged in the vertical and horizontal directions. It means that the 2-dof stage includes the four modules of the 1-dof stage. The positioning stage is employed to locate the biomaterials in an in situ nanoindentation system. As illustrated in Figure 2(a), the P-joint shows a leaf hinge, so-called as the prismatic joint, to generate a large deformation. Meanwhile, the remain positions are designed with the elliptical hinges to ensure good positions. The overall working operation of the 2-dof stage is mainly based on the deformations of the elliptical joints, right circular joints, and P-joints. The suggested stage is driven using a PZT actuator. The main dimensioning parameters of the proposed stage are illustrated in Figure 2(b). The values of the parameters are provided in Table 1.

In the proposed design, a double-lever amplification mechanism is widely used for many researches on flexure-based mechanisms [16]. Meanwhile, the symmetric eight-lever displacement amplifier integrated into the 2-dof stage for locating biospecimens has been investigated. Therefore, the proposed displacement amplifier is a new structure for positioning a biomaterial sample in in situ nanoindentation.

Each component has an important task in overall nanoindentation. Specifically, the main task of the 2-dof stage is aimed to locate the fine position of the biospecimens. The only difference of the proposed mechanism design in nanoindentation/in situ nanoindentation in comparison with traditional mechanisms is a monolithic structure in order to reduce the mechanical component quantity as well as to reduce friction and wear of an assembly cluster. Therefore, inspired from the advantages of the compliant mechanisms, the authors will investigate to contribute for the new compliant structures.

As illustrated clearly in Figure 2(b) and Table 1, the parameters *A*, *B*, and *C* with different colors are the thicknesses of the elliptical hinges at lever floor 1, lever floor 2, and lever floors 3 and 4, respectively. In addition, *D* is the thickness of the right circular hinge at the end of lever floor 4, and *E* is the thickness of the leaf hinge of the parallel guiding mechanism. In addition, the technical specifications of the proposed stage are determined in Table 2.

## 3. Proposed Methodology

In this part, a modeling and dimensional optimization synthesis method is developed for the compliant 2-dof stage. The proposed methodology is formed based on the analytical methods and the metaheuristic algorithm. In this work, the deformable mechanics theory is coupled with the elastic beam theory and Lagrange method in establishing the kinetostatic and dymamic equations of the 2-dof stage.

### 3.1. Modeling and Dimensional Optimization Synthesis

**Figure 3:**
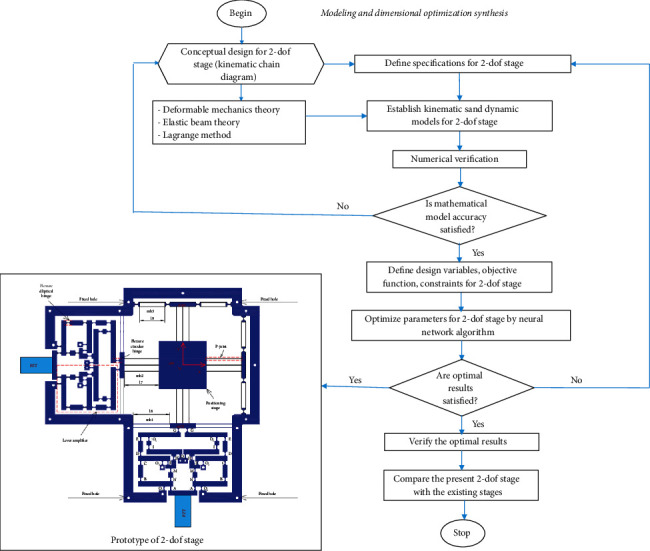
Flowchart of the proposed methodology for modeling and optimization synthesis of the 2-dof stage.

The flowchart of the proposed methodology for modeling and dimensional optimization synthesis for the developed stage is shown in Figure 3. The main steps of the present methodology are briefly performed as follows:

- Step 1: predetermine a conceptual design of the 2-dof stage, i.e., a built kinematic chain diagram.
- Step 2: predetermine the specification's requirements for the 2-dof stage.
- Step 3: establish the kinetostatic and dynamic mathematical equations for the 2-dof stage by using the the deformable mechanics theory, the elastic beam theory, and the Lagrange approach.
- Step 4: verify the corrections of mathematical equations through finite element analysis (FEA).
- Step 5: if the kinematic and dynamic equations are corrected, the calculation process moves to the further steps. Otherwise, the process goes back the step 1.
- Step 6: define the design variables, objective, and constraint functions for the 2-dof stage.
- Step 7: in order to optimize the parameters of the 2-dof stage, the neural network algorithm is employed in improving the dynamic performance of the stage.
- Step 8: the optimal results are verified via FEA analysis. If it satisfied, a protoype of the stage will be made.
- Step 9: the dynamic performance of the 2-dof stage is compared with that from the previous stages.

### 3.2. Neural Network Algorithm

**Figure 4:**
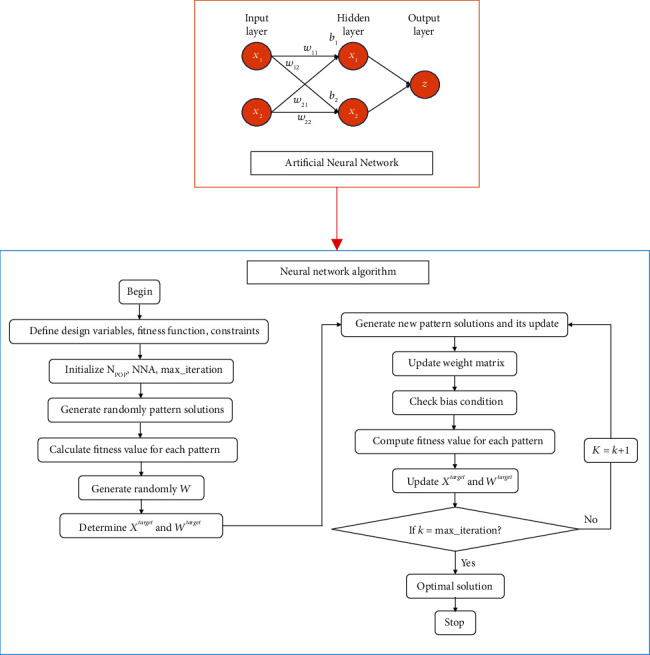
Schematic diagram of the neural network algorithm.

Inspired from the artificial neural network (ANN), the neural network algorithm (NNA) was developed [17]. This NNA optimizer is also considered as other metaheuristic optimizers based on the population differential evolution algorithm [18], particle swarm optimization [19], nondominated sorting genetic algorithm [20], and so on. This algorithm is aimed to decrease the error among the targets and the predicted values, and achieve a global optimally best result. The flowchart of the NNA is given in Figure 4.

A pattern solution (*P*_s_) is determined as

(1) $$\begin{array}{c} P_{s}=\left[x_{1},x_{2},\ldots ,x_{d}\right],\;d:\text{problem size}. \end{array}$$

A matrix of pattern solution (*X*) is defined by

(2) $$\begin{array}{c} X=\left[\begin{array}{ccc} x_{1}^{1} & x_{2}^{1} & x_{D}^{1}\\ \vdots & \ddots & \vdots \\ x_{1}^{npop} & x_{2}^{npop} & x_{D}^{npop} \end{array}\right],\text{ npop is the population number.} \end{array}$$

The *i* ^th^ cost of the function is defined as follows:

(3) $$\begin{array}{c} C_{i}=f\left(x_{1}^{i},x_{2}^{i},\ldots ,x_{D}^{i}\right). \end{array}$$

A weight matrix is determined by

(4) $$\begin{array}{c} W\left(t\right)=\left[\begin{array}{c} W_{1}^{1}\;W_{2}^{i}\;\cdots \;W_{D}^{npop}\\ \;\vdots \ddots \;\vdots \;\\ W_{npop}^{1}\;W_{npop}^{i}\cdots W_{npop}^{npop} \end{array}\right]=\left[\begin{array}{c} W_{11}\;\cdots \;W_{i1}\;\cdots \;W_{npop1}\\ \;\vdots \;\ddots \;\vdots \;\\ W_{1\;npop}\;W_{i\;npop}\;\cdots \;W_{npop\;npop} \end{array}\right]. \end{array}$$

The weight value is determined as

(5) $$\begin{array}{c} \sum_{j=1}^{npop}w_{ij}\left(t\right)=1,\;i\in npop, \end{array}$$

(6) $$\begin{array}{c} w_{ij}\in U\left(0,1\right),\;i,j\in \;npop, \end{array}$$

A new matrix (*X*) is calculated by

(7) $$\begin{array}{c} X_{j}^{new}\left(t+1\right)=\sum_{t=1}^{npop}w_{ij}\left(t\right)\times X_{t}\left(t\right),\;j\;\in \;npop, \end{array}$$

(8) $$\begin{array}{c} X_{i}\left(t+1\right)=X_{i}\left(t\right)+X_{i}^{new}\left(t+1\right),\;i\;\in \;npop. \end{array}$$

The weight matrix is updated by following formula:

(9) $$\begin{array}{c} W_{t}^{\text{update}}\left(t+1\right)=W_{i}\left(t\right)+2\times rand\times \left(W^{\text{target}}\left(t\right)-W_{i}\left(t\right)\right),\;i\;\in \;nppop, \end{array}$$

A bias means that the exploration influences a new solution and weighting. Considering *rand* > *ψ* (*ψ* is solutions percentage), the function operator is computed by

(10) $$\begin{array}{c} X_{t}^{*}\left(t+1\right)=X_{i}\left(t+1\right)+2\times rand\times \left(X^{\text{target}}\left(t\right)-X_{i}\left(t+1\right)\right),\;i\;\in \;nppop, \end{array}$$

In this work, an initial population of 20, max iterations of 500, and *ψ* of 1 are utilized for the NNA.

## 4. Results and Discussion

### 4.1. Modeling of Kinetostatics and Dynamics

The kinetostatics and dynamics of the 2-dof stage are performed to evaluate its behaviors. The deformable mechanics theory, the elastic beam theory, and the Lagrange method are utilized for these analyses. In this work, the specifications of the 2-dof stage are analyzed (i.e., ratio of displacement amplification, stiffness, stroke, and resonant frequency).

#### 4.1.1. Modeling of Amplification Ratio and Input Stiffness

**Figure 5:**
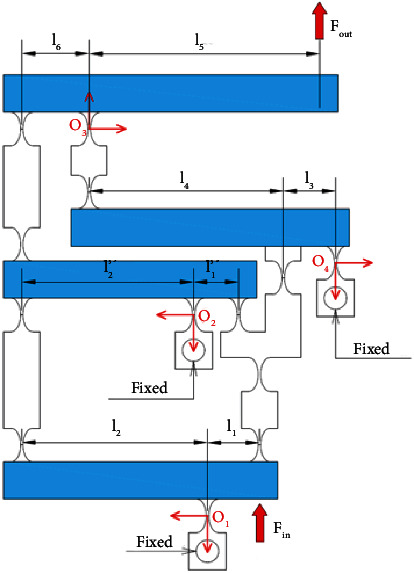
A variant of the symmetrical displacement lever amplifier.

Figure 5 demonstrates a variant of the modified displacement lever amplifier (MDLA), a so-called variant of a lever amplifier. It includes the main parameters of the lever lengths of the different amplification floors. Because of a symmetrical structure, a half of the displacement amplifier is chosen to analyze the quality characteristics of the proposed 2-dof stage.

**Figure 6:**
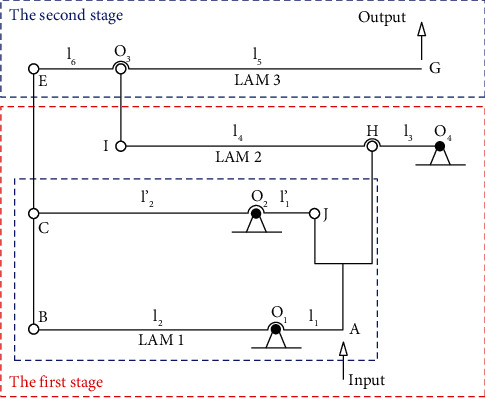
Schematic diagram of the modified displacement lever amplifier.

A schematic diagram of the MDLA is provided in Figure 6. It consists of main levers, including the lever amplification mechanism #1 (LAM1), lever amplification mechanism #2 (LAM2), and lever amplification mechanism #3 (LAM3). Besides, the parameters of the lengths of levers (*l*_*1*_, *l*_*2*_, *l*_*1*_′, *l*_*2*_′, *l*_*3*_, *l*_*4*_, *l*_*5*_, and *l*_*6*_) are also in Figure 6 in which O_1_, O_2_, O_3_, and O_4_ are the rotation centers of the levers.

As shown in Figure 6, the MDLA is operated based on three levers (LAM1, LAM2, and LAM3). It can be determined that the output displacement of the amplifier can be derived as follows [21]:

(11) $$\begin{array}{c} \delta_{out}=\left[\frac{l_{5}}{l_{6}}\left(\frac{l_{2}}{l_{1}}+\frac{l_{4}}{l_{3}}+1\right)+\frac{l_{4}}{l_{3}}+1\right]\delta_{in}, \end{array}$$

where *δ*_*in*_ and *δ*_*out*_ symbolize the input displacement and the output displacement, respectively. Next, the amplification ratio will be computed as follows:

(12) $$\begin{array}{c} A=\frac{l_{5}}{l_{6}}\left(\frac{l_{2}}{l_{1}}+\frac{l_{4}}{l_{3}}+1\right)+\frac{l_{4}}{l_{3}}+1, \end{array}$$

where *A* is the displacement amplification ratio.

**Figure 7:**
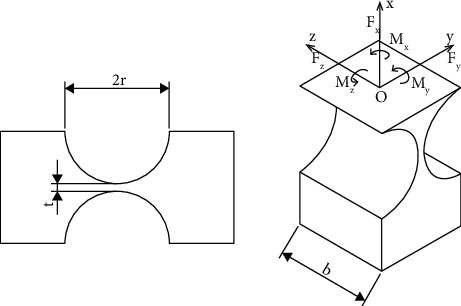
Diagram of a circular arc flexure hinge.

Three kinds of flexure joints are used at appropriate places in the original design, thus helping the design to achieve a large displacement and reduce undesirable motions of the stage.  Figure 7 demonstrates the main associated dimensions of a flexure circular notched hinge, and it shows the effect of force and torque that recovers when the mechanism operates.

The mechanism's input stiffness is investigated. The general spring with bending stiffness (*K*_*δxFx*_^*c*^) is generated via the lateral force, and the torsion stiffness (*K*_*θzMz*_^*c*^) is made via the torque. The linear stiffness (*K*_*θyMy*_^*c*^) is created via the axial force for the flexure right circular hinge. Furthermore, the leaf beam's torsion and linear stiffness are denoted by *K*_*θzMz*_^*l*^ and *K*_*θyMy*_^*l*^, respectively. Correspondingly, the flexure elliptical notched hinge is behaved with bending stiffness (*K*_*δ*_*X*_ *F*_*X*__^*e*^) produced via the lateral force, torsion stiffness (*K* ^*e*^ _*θzMz*_) produced via the torque, and linear stiffness (*K*_*θyMy*_^*e*^) generated via the axial force [22].

(13) $$\begin{array}{c} K_{\theta zMz}^{c}=\frac{2Ebt^{2.5}}{9\pi r^{0.5}}, \end{array}$$

(14) $$\begin{array}{c} K_{\theta yMy}^{c}=\frac{Ebt^{0.5}}{\pi \left(r^{0.5}-0.5t^{0.5}\right)}, \end{array}$$

(15) $$\begin{array}{c} K_{\delta xFx}^{c}=\frac{2Ebt^{2.5}}{3\pi r^{1.5}\left(3r+t\right)}, \end{array}$$

**Figure 8:**
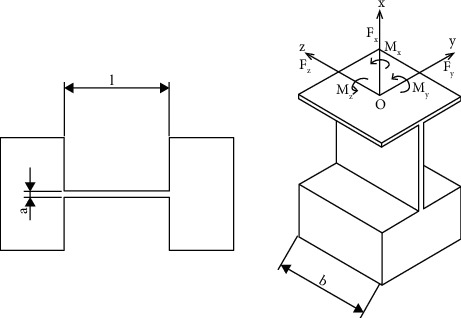
Diagram of a leaf-type arc flexure hinge.

Next, Figure 8 describes the simplified structure diagram of the flexure leaf hinge. In addition, the main parameters such as the length, width, and thickness are also displayed along with the direction of force and torque acting on the leaf hinge.

Stiffness values of the leaf hinge are calculated with the following equations:

(16) $$\begin{array}{c} K_{\theta zMz}^{l}=\frac{Eba^{3}}{12l}, \end{array}$$

(17) $$\begin{array}{c} K_{\theta yMy}^{l}=\frac{Eba}{l}, \end{array}$$

(18) $$\begin{array}{c} M_{\theta zMz}^{l}=K_{\theta zMz}^{l}\Delta \theta . \end{array}$$

**Figure 9:**
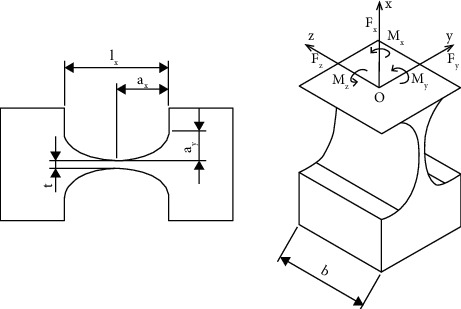
Diagram of an elliptical arc flexure hinge.

Figure 9 shows the dimensions of the flexure elliptical hinge as well as the directions of the force and moment acting on the elliptical hinge.

Next, the following equations describe how to calculate the stiffness of the flexure elliptical hinge that bends in different directions.

(19) $$\begin{array}{c} K^{e}{\;}_{\theta zMz}=\frac{2Eba_{X}^{2}}{3\in f\left(\beta_{y}\right)}, \end{array}$$

(20) $$\begin{array}{c} K_{\theta yMy}^{e}=\frac{Eb^{3}}{12\in g\left(\beta_{y}\right)}, \end{array}$$

(21) $$\begin{array}{c} K_{\delta_{X}F_{X}}^{e}=\frac{Eb}{\in g\left(\beta_{y}\right)}, \end{array}$$

(22) $$\begin{array}{c} \beta_{X}=\frac{t}{2a_{X}}, \end{array}$$

(23) $$\begin{array}{c} \beta_{y}=\frac{t}{2a_{y}}, \end{array}$$

(24) $$\begin{array}{c} \in =\frac{a_{X}}{a_{y}}=\frac{\beta_{y}}{\beta_{x}}, \end{array}$$

(25) $$\begin{array}{c} f\left(\beta_{y}\right)=f\left(\in \beta_{x}\right)=\frac{1}{2\beta_{y}+\beta_{y}^{2}}\left(\frac{3+4\beta_{y}+2\beta_{y}^{2}}{\left(1+\beta_{y}\right)\left(2\beta_{y}+\beta_{y}^{2}\right)}+\frac{6\left(1+\beta_{y}\right)}{{\left(\sqrt{2\beta_{y}+\beta_{y}^{2}}\right)}^{3}}\tan^{-1}\sqrt{\frac{2+\beta_{y}}{\beta_{y}}}\right), \end{array}$$

(26) $$\begin{array}{c} g\left(\beta_{y}\right)=g\left(\in \beta_{x}\right)=\frac{2\left(1+\beta_{y}\right)}{\sqrt{2\beta_{y}+\beta_{y}^{2}}}\tan^{-1}\sqrt{\frac{2+\beta_{y}}{\beta_{y}}}-\frac{\pi }{2}, \end{array}$$

where *b* denotes the thickness of the flexure hinge, *E* denotes the elastic modulus of the manufacturing material, *r* is the radius of a flexure circular notched hinge, and *t* denotes the smallest width of the circular/elliptical hinge. The width and length of the leaf beam are represented by *a* and *l,* respectively. The dimensionless factor representing the elliptical hinge geometry *a*_*x*_ is denoted by (*β*_*X*_), and (*a*_*y*_) is denoted by (*β*_*y*_). The symbol (∈) is the multiplication factor of the ratio of major to minor axes. On the other hand, the symbols (*f*(*β*_*x*_)) and (*f*(*β*_*y*_)) are the dimensionless compliance factors according to (*β*_*X*_) and (*β*_*y*_), respectively.

By subjecting an input displacement (∈) at the stage's input end, the corresponding input force (*F*_*in*_) is determined via the following equation:

(27) $$\begin{array}{c} F_{in}=K_{in}\delta_{in}, \end{array}$$

where *K*_*in*_ is the input stiffness of the micromanipulator.

**Figure 10:**
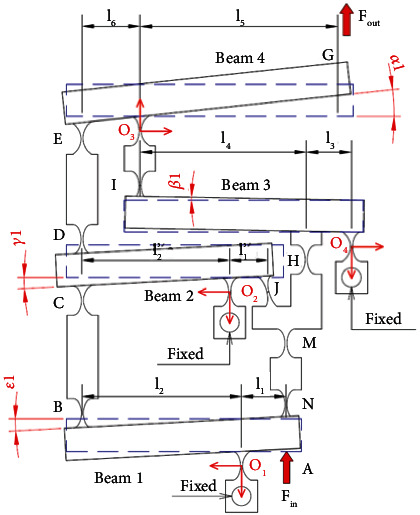
Force and deformation diagram of a half of the MDLA.

Figure 10 describes the initial state and working state of the MDLA. The deformation angles formed from the position change of the multistage lever help to show the different degrees of deformation of the structure while it is working.

**Figure 11:**
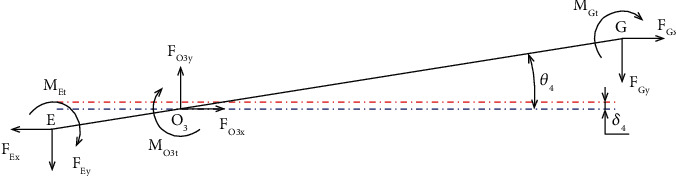
Force analysis diagram of beam 4.

**Figure 12 fig12:**
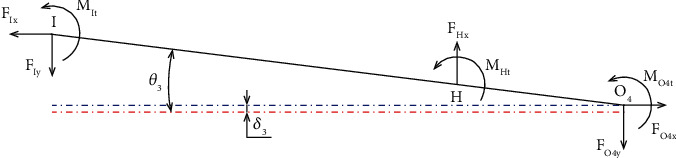
Force analysis diagram of beam 3.

**Figure 13 fig13:**
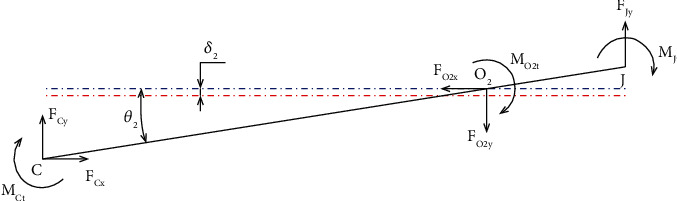
Force analysis diagram of beam 2.

**Figure 14:**
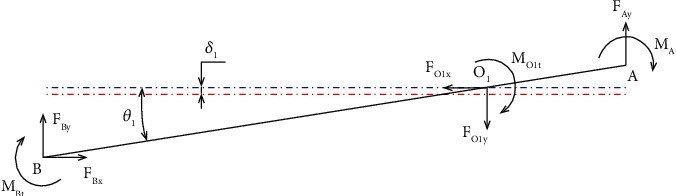
Force analysis diagram of beam 1.

Figures 11 [Fig fig12] [Fig fig13]–14 present the force analysis diagram of beam 4, beam 3, beam 2, and beam 1, respectively, in the lever amplification mechanism. The diagrams illustrate the details of the applied force, moment, rotation angle, as well as the deformation of the elastic joint.

Only half of the displacement amplification structure is investigated due to the symmetrical structure. A main force analysis scheme of the mechanical amplification mechanism is depicted in Figure 10. As a result, the following equations are formed by using the mechanic equilibrium state of beam 4, as asserted in Figure 11.

The following force equilibrium equations can be derived as

(28) $$\begin{array}{c} F_{O3y}=F_{Ey}+F_{Gy}, \end{array}$$

(29) $$\begin{array}{c} F_{Ey}l_{6}=F_{Gy}l_{5}+M_{O3t}+M_{Gt}+M_{Et}. \end{array}$$

Specifically, the bending moments of the points O_3_, G, and E are represented as *M*_*O3t*,_ *M*_*Gt*,_, and *M*_*Et*_, respectively. Three forces *F*_*O*3*y*_, *F*_*Ey*_, and *F*_*Gy*_ are acting at points O_3_, *E*, and G in the direction of *y*-axis when the structure operates, respectively. The displacement value at point G is a combination of two displacements including displacement due to hinges at O_3_ rotating around its axis (*l*_*5*_ *θ*_*4*_) and displacement due to drift (*δ*_4_). Furthermore, by multiplying stiffness by displacement, the forces *F*_*O3y*_ and *F*_*Gy*_ are determined. They are calculated via the following equations:

(30) $$\begin{array}{c} F_{O3y}=K_{O3x}\delta_{4}, \end{array}$$

(31) $$\begin{array}{c} F_{Gy}=K_{Gy}\left(l_{5}\theta_{4}-\delta_{4}\right), \end{array}$$

(32) $$\begin{array}{c} M_{O3t}=K_{O3t}\theta_{4}, \end{array}$$

(33) $$\begin{array}{c} M_{Gt}=K_{Gt}\theta_{4}, \end{array}$$

(34) $$\begin{array}{c} M_{Et}=K_{Et}\theta_{4}, \end{array}$$

where *K*_*O3x*_ is the lateral bending stiffness by the force *F*_*O3y*_, and *K*_*Gl*_ is the G point's output stiffness of the MDLA. In addition, the rotational stiffnesses *K*_*O3t*_, *K*_*Gt*_, and *K*_*Et*_ are the stiffnesses of the flexure hinges at points O_3_, G, and *E*, respectively.

Substituting (30) and (31) into (28), the results are yielded as

(35) $$\begin{array}{c} K_{O3x}\delta_{4}=F_{Ey}+K_{Gy}\left(l_{5}\theta_{4}-\delta_{4}\right), \end{array}$$

(36) $$\begin{array}{c} \delta_{4}=\frac{F_{Ey}+K_{Gy}l_{5}\theta_{4}}{K_{O3x}+K_{Gy}}. \end{array}$$

Substituting (31)–(34) into (29), the results are achieved as

(37a) $$\begin{array}{c} F_{Ey}l_{6}=K_{Gy}l_{5}\left(l_{5}\theta_{4}-\delta_{4}\right)+K_{O3t}\theta_{4}+K_{Gt}\theta_{4}+K_{Et}\theta_{4}, \end{array}$$

(37b) $$\begin{array}{c} F_{Ey}l_{6}+K_{Gy}l_{5}\delta_{4}=\left(K_{Gy}l_{5}^{2}+K_{O3t}+K_{Gt}+K_{Et}\right)\theta_{4}, \end{array}$$

where *F*_*Ey*_, *F*_*O3y*_, and *F*_*Gy*_ are the forces generated at points of *E*, O_3_, and G, respectively, when the structure operates.

Substituting (36) into (37b), the *θ*_4_ and *δ*_4_ can be derived as

(38) $$\begin{array}{c} F_{Ey}l_{6}+K_{Gy}l_{5}\frac{F_{Ey}+K_{Gy}l_{5}\theta_{4}}{K_{O3x}+K_{Gy}}=\left(K_{Gy}l_{5}^{2}+K_{O3t}+K_{Gt}+K_{Et}\right)\theta_{4}, \end{array}$$

(39) $$\begin{array}{c} F_{Ey}l_{6}\left(K_{O3x}+K_{Gy}\right)+K_{Gy}l_{5}\left(F_{Ey}+K_{Gy}l_{5}\theta_{4}\right)=\left(K_{O3x}+K_{Gy}\right)\left(K_{Gy}l_{5}^{2}+K_{O3t}+K_{Gt}+K_{Et}\right)\theta_{4}, \end{array}$$

(40) $$\begin{array}{c} \theta_{4}=\frac{l_{6}\left(K_{O3x}+K_{Gy}\right)+K_{Gy}l_{5}}{\left(K_{O3x}+K_{Gy}\right)\left(K_{Gy}l_{5}^{2}+K_{O3t}+K_{Gt}+K_{Et}\right)-K_{Gy}^{2}l_{5}^{2}}F_{Ey}, \end{array}$$

(41a) $$\begin{array}{c} \delta_{4}=\frac{F_{Ey}+K_{Gy}l_{5}l_{6}\left(K_{O3x}+K_{Gy}\right)+K_{Gy}l_{5}/\left(K_{O3x}+K_{Gy}\right)\left(K_{Gy}l_{5}^{2}+K_{O3t}+K_{Gt}+K_{Et}\right)-K_{Gy}^{2}l_{5}^{2}F_{Ey}}{K_{O3x}+K_{Gy}}, \end{array}$$

(41b) $$\begin{array}{c} \delta_{4}=\frac{\left(K_{O3x}+K_{Gy}\right)\left(K_{Gy}l_{5}^{2}+K_{O3t}+K_{Gt}+K_{Et}\right)-K_{Gy}^{2}l_{5}^{2}+K_{Gy}l_{5}\left(l_{6}\left(K_{O3x}+K_{Gy}\right)+K_{Gy}l_{5}\right)}{\left(K_{O3x}+K_{Gy}\right)\left[\left(K_{O3x}+K_{Gy}\right)\left(K_{Gy}l_{5}^{2}+K_{O3t}+K_{Gt}+K_{Et}\right)-K_{Gy}^{2}l_{5}^{2}\right]}F_{Ey}, \end{array}$$

(41c) $$\begin{array}{c} \delta_{4}=\frac{K_{Gy}l_{5}^{2}+K_{O3t}+K_{Gt}+K_{Et}+K_{Gy}l_{5}l_{6}}{\left(K_{O3x}+K_{Gy}\right)\left(K_{Gy}l_{5}^{2}+K_{O3t}+K_{Gt}+K_{Et}\right)-K_{Gy}^{2}l_{5}^{2}}F_{Ey}. \end{array}$$

Regarding beam 4, the ratio of the amplifier and the input stiffness are determined by

(42) $$\begin{array}{c} \lambda_{4}=\frac{l_{5}\theta_{4}-\delta_{4}}{l_{6}\theta_{4}+\delta_{4}}, \end{array}$$

(43) $$\begin{array}{c} K_{in4}=\frac{F_{Ey}}{l_{6}\theta_{4}+\delta_{4}}. \end{array}$$

Substituting (40) and (40c) into (42) and (43), respectively, the following formulas can be obtained:

(44a) $$\begin{array}{c} \lambda_{4}=\frac{l_{5}\left[l_{6}\left(K_{O3x}+K_{Gy}\right)+K_{Gy}l_{5}\right]-\left(K_{Gy}l_{5}^{2}+K_{O3t}+K_{Gt}+K_{Et}+K_{Gy}l_{5}l_{6}\right)}{l_{6}\left[l_{6}\left(K_{O3x}+K_{Gy}\right)+K_{Gy}l_{5}\right]+\left(K_{Gy}l_{5}^{2}+K_{O3t}+K_{Gt}+K_{Et}+K_{Gy}l_{5}l_{6}\right)}, \end{array}$$

(44b) $$\begin{array}{c} \lambda_{4}=\frac{l_{5}l_{6}\left(K_{O3x}+K_{Gy}\right)-\left(K_{O3t}+K_{Gt}+K_{Et}+K_{Gy}l_{5}l_{6}\right)}{l_{6}\left(K_{O3x}+K_{Gy}\right)+2K_{Gy}l_{5}l_{6}+K_{Gy}l_{5}^{2}+K_{O3t}+K_{Gt}+K_{Et}}, \end{array}$$

(44c) $$\begin{array}{c} \lambda_{4}=\frac{l_{5}l_{6}K_{O3x}-K_{O3t}-K_{Gt}-K_{Et}}{K_{Gy}{\;}_{\left(l_{5}+l_{6}\right)}^{2}+K_{O3x}l_{6}^{2}+K_{O3t}+K_{Gt}+K_{Et}}, \end{array}$$

(45) $$\begin{array}{c} K_{in4}=\frac{\left(K_{O3x}+K_{Gy}\right)\left(K_{Gy}l_{5}^{2}+K_{O3t}+K_{Gt}+K_{Et}\right)-K_{Gy}^{2}l_{5}^{2}}{K_{Gy}{\;}_{\left(l_{5}+l_{6}\right)}^{2}+K_{O3x}l_{6}^{2}+K_{O3t}+K_{Gt}+K_{Et}}. \end{array}$$

Figure 12 presents the force analysis diagram of beam 3.

Considering the equilibrium state of beam 3, the force and moment are computed as

(46) $$\begin{array}{c} F_{Hy}=F_{Iy}+F_{O4y}, \end{array}$$

(47) $$\begin{array}{c} F_{Hy}l_{3}=F_{Iy}\left(l_{3}+l_{4}\right)+M_{It}+M_{Ht}+M_{O4t}, \end{array}$$

where *M*_*O4t*_, *M*_*It*,_, and *M*_*Ht*_ are moments of bending of the points O_4_, *I*, and *H*. Moreover, *F*_*O*4*y*_, *F*_*Hy*_, and *F*_*Iy*_ are forces which are acting at point O_4_, H, and I in the direction of *y*-axis, respectively.

The displacement value at point I is a combination of two displacements including displacement due to hinges at O_4_ rotating around its axis ((*l*_*3*_ + *l*_*4*_) *θ*_*3*_) and displacement due to drift (*δ*_3_).

Furthermore, the forces *F*_*O4y*_ and *F*_*Iy*_ can be calculated as

(48) $$\begin{array}{c} F_{O4y}=K_{O4x}\delta_{3}, \end{array}$$

(49) $$\begin{array}{c} F_{Iy}=K_{3}\left[\left(l_{3}+l_{4}\right)\theta_{3}+\delta_{3}\right], \end{array}$$

(50) $$\begin{array}{c} M_{O4t}=K_{O4t}\theta_{3}, \end{array}$$

(51) $$\begin{array}{c} M_{It}=K_{It}\theta_{3}, \end{array}$$

(52) $$\begin{array}{c} M_{Ht}=K_{Ht}\theta_{3}, \end{array}$$

where *K*_*O4x*_ is the lateral bending stiffness by force *F*_*O4y*_, and *K*_*3*_ is the I point's output stiffness. *K*_*O4t*_ is the O_4_ point's rotational stiffness, and *K*_*It*_, and *K*_*Ht*_ are the rotational stiffness of I and H, respectively.

Substituting (48) and (49) into (46), the results are obtained as

(53) $$\begin{array}{c} F_{Hy}=K_{3}\left(\left(l_{3}+l_{4}\right)\theta_{3}+\delta_{3}\right)+K_{O4x}\delta_{3}, \end{array}$$

(54) $$\begin{array}{c} \delta_{3}=\frac{F_{Hy}-K_{3}\left(l_{3}+l_{4}\right)\theta_{3}}{K_{O4x}+K_{3}}. \end{array}$$

Substituting (49)–(52) into (47), the results are yielded as

(55a) $$\begin{array}{c} F_{Hy}l_{3}=K_{3}\left(l_{3}+l_{4}\right)\left(\left(l_{3}+l_{4}\right)\theta_{3}+\delta_{3}\right)+K_{O4t}\theta_{3}+K_{It}\theta_{3}+K_{Ht}\theta_{3}, \end{array}$$

(55b) $$\begin{array}{c} F_{Hy}l_{3}=K_{3}\left(l_{3}+l_{4}\right)\delta_{3}+\left(K_{3}{\;}_{\left(l_{3}+l_{4}\right)}^{2}+K_{O4t}+K_{It}+K_{Ht}\right)\theta_{3}. \end{array}$$

Substituting (54) into (55b) *θ*_3_ and *δ*_3_ can be derived as

(56a) $$\begin{array}{c} F_{Hy}l_{3}=K_{3}\left(l_{3}+l_{4}\right)\frac{F_{Hy}-K_{3}\left(l_{3}+l_{4}\right)\theta_{3}}{K_{O4x}+K_{3}}+\left[K_{3}{\;}_{\left(l_{3}+l_{4}\right)}^{2}+K_{O4t}+K_{It}+K_{Ht}\right]\theta_{3}, \end{array}$$

(56b) $$\begin{array}{c} F_{Hy}l_{3}\left(K_{O4x}+K_{3}\right)-K_{3}\left(l_{3}+l_{4}\right)F_{Hy}=\left[\left(K_{O4x}+K_{3}\right)\left[K_{3}{\left(l_{3}+l_{4}\right)}^{2}+K_{O4t}+K_{It}+K_{Ht}\right]-K_{3}^{2}{\left(l_{3}+l_{4}\right)}^{2}\right]\theta_{3}, \end{array}$$

(57) $$\begin{array}{c} \theta_{3}=\frac{l_{3}\left(K_{O4x}+K_{3}\right)-K_{3}\left(l_{3}+l_{4}\right)}{\left(K_{O4x}+K_{3}\right)\left[K_{3}{\left(l_{3}+l_{4}\right)}^{2}+K_{O4t}+K_{It}+K_{Ht}\right]-K_{3}{\;}^{2}{\left(l_{3}+l_{4}\right)}^{2}}F_{Hy}, \end{array}$$

(58a) $$\begin{array}{c} \delta_{3}=\frac{F_{Hy}-K_{3}\left(l_{3}+l_{4}\right)l_{3}\left(K_{O4x}+K_{3}\right)-K_{3}\left(l_{3}+l_{4}\right)/\left(K_{O4x}+K_{3}\right)\left[K_{3}{\left(l_{3}+l_{4}\right)}^{2}+K_{O4t}+K_{It}+K_{Ht}\right]-K_{3}^{2}{\left(l_{3}+l_{4}\right)}^{2}F_{Hy}}{K_{O4x}+K_{3}}, \end{array}$$

(58b) $$\begin{array}{c} \delta_{3}=\frac{K_{3}{\left(l_{3}+l_{4}\right)}^{2}-K_{3}l_{3}+K_{O4t}+K_{It}+K_{Ht}}{\left(K_{O4x}+K_{3}\right)\left[K_{3}{\left(l_{3}+l_{4}\right)}^{2}+K_{O4t}+K_{It}+K_{Ht}\right]-K_{3}^{2}{\left(l_{3}+l_{4}\right)}^{2}}F_{Hy}. \end{array}$$

Considering beam 3, the ratio of the amplifier and the input stiffness are expressed by

(59) $$\begin{array}{c} \lambda_{3}=\frac{\left(l_{3}+l_{4}\right)\theta_{3}+\delta_{3}}{l_{3}\theta_{3}+\delta_{3}}, \end{array}$$

(60) $$\begin{array}{c} K_{in3}=\frac{F_{Hy}}{l_{3}\theta_{3}+\delta_{3}}. \end{array}$$

Substituting (57) and (58b) into (59) and (60), respectively, the following formulas can be obtained:

(61a) $$\begin{array}{c} \lambda_{3}=\frac{\left(l_{3}+l_{4}\right)\left[l_{3}\left(K_{O4x}+K_{3}\right)-K_{3}\left(l_{3}+l_{4}\right)\right]+K_{3}{\left(l_{3}+l_{4}\right)}^{2}-K_{3}l_{3}+K_{O4t}+K_{It}+K_{Ht}}{l_{3}\left[l_{3}\left(K_{O4x}+K_{3}\right)-K_{3}\left(l_{3}+l_{4}\right)\right]+K_{3}{\left(l_{3}+l_{4}\right)}^{2}-K_{3}l_{3}+K_{O4t}+K_{It}+K_{Ht}}, \end{array}$$

(61b) $$\begin{array}{c} \lambda_{3}=\frac{l_{3}\left(K_{O4x}+K_{3}\right)\left(l_{3}+l_{4}\right)-K_{3}{\left(l_{3}+l_{4}\right)}^{2}+K_{3}{\;}_{\left(l_{3}+l_{4}\right)}^{2}-K_{3}l_{3}+K_{O4t}+K_{It}+K_{Ht}}{l_{3}^{2}\left(K_{O4x}+K_{3}\right)-K_{3}l_{3}\left(l_{3}+l_{4}\right)+K_{3}{\left(l_{3}+l_{4}\right)}^{2}-K_{3}l_{3}+K_{O4t}+K_{It}+K_{Ht}}, \end{array}$$

(61c) $$\begin{array}{c} \lambda_{3}=\frac{l_{3}\left(K_{O4x}+K_{3}\right)\left(l_{3}+l_{4}\right)-K_{3}l_{3}+K_{O4t}+K_{It}+K_{Ht}}{l_{3}^{2}\left(K_{O4x}+K_{3}\right)-K_{3}l_{3}\left(l_{3}+l_{4}\right)+K_{3}{\left(l_{3}+l_{4}\right)}^{2}-K_{3}l_{3}+K_{O4t}+K_{It}+K_{Ht}}, \end{array}$$

(62) $$\begin{array}{c} K_{in3}=\frac{\left(K_{O4x}+K_{3}\right)\left(K_{3}{\left(l_{3}+l_{4}\right)}^{2}+K_{O4t}+K_{It}+K_{Ht}\right)-K_{3}^{2}{\left(l_{3}+l_{4}\right)}^{2}}{l_{3}^{2}\left(K_{O4x}+K_{3}\right)-K_{3}l_{3}\left(l_{3}+l_{4}\right)+K_{3}{\left(l_{3}+l_{4}\right)}^{2}-K_{3}l_{3}+K_{O4t}+K_{It}+K_{Ht}}, \end{array}$$

(63) $$\begin{array}{c} K_{3}=\frac{K_{O3y}K_{in4}}{K_{O3y}+K_{in4}}. \end{array}$$

Figure 13 shows the force analysis diagram of beam 2.

In a similar calculation for beam 2, the forces and moments are computed by

(64) $$\begin{array}{c} F_{O2y}=F_{Cy}+F_{Jy}, \end{array}$$

(65) $$\begin{array}{c} F_{Jy}l_{1}^{\prime }=F_{Cy}l_{2}^{\prime }+M_{O2t}+M_{Jt}+M_{Ct}, \end{array}$$

where *M*_*O2t*_, *M*_*Jt*_, and *M*_*Ct*_ are bending moments at the O_2_, *J*, and C points, respectively. The forces acting at points O_2_, C, and *J* in the direction of *y*-axis are *F*_*O*2*y*_, *F*_*Cy*_, and *F*_*Jy*_, respectively.

The displacement value at point C is a combination of two displacements including displacement due to hinges at O_2_ rotating around its axis (*l'*_*2*_ *θ*_*2*_) and displacement due to drift (*δ*_2_).

Moreover, the forces *F*_*O2y*_ and *F*_*Cy*_ are expressed as

(66) $$\begin{array}{c} F_{O2y}=K_{O2x}\delta_{2}, \end{array}$$

(67) $$\begin{array}{c} F_{Cy}=K_{2}\left(l_{2}^{\prime }\theta_{2}-\delta_{2}\right), \end{array}$$

(68) $$\begin{array}{c} M_{O2t}=K_{O2t}\theta_{2}, \end{array}$$

(69) $$\begin{array}{c} M_{Ct}=K_{Ct}\theta_{2}, \end{array}$$

(70) $$\begin{array}{c} M_{Jt}=K_{Jt}\theta_{2}, \end{array}$$

where *K*_*O2x*_ is the lateral bending stiffness by force *F*_*O2y*_, and *K*_*2*_ is the output stiffness of the *E* point. *K*_*O2t*_ is the rotational stiffness of the O_2_ point. *K*_*Jt*_ and *K*_*Ct*_ are the rotational stiffnesses of *J*, and C points, respectively. Substituting (66) and (67) into (64),

(71) $$\begin{array}{c} K_{O2x}\delta_{2}=F_{Jy}+K_{2}\left(l_{2}^{\prime }\theta_{2}-\delta_{2}\right), \end{array}$$

(72) $$\begin{array}{c} \delta_{2}=\frac{F_{Jy}+K_{2}l_{2}^{\prime }\theta_{2}}{K_{O2x}+K_{2}}. \end{array}$$

Substituting (67)–(70) into (65), the following equations can be derived:

(73a) $$\begin{array}{c} F_{Jy}l_{1}^{\prime }=K_{2}l_{2}^{\prime }\left(l_{2}^{\prime }\theta_{2}-\delta_{2}\right)+K_{O2t}\theta_{2}+K_{Ct}\theta_{2}+K_{Jt}\theta_{2}, \end{array}$$

(73b) $$\begin{array}{c} F_{Jy}l_{1}^{\prime }+K_{2}l_{2}^{\prime }\delta_{2}=\left(K_{2}l_{2}^{\prime 2}+K_{O2t}+K_{Ct}+K_{Jt}\right)\theta_{2}. \end{array}$$

Substituting (72) into (73b), the value of *δ*_2_ can be derived as

(74a) $$\begin{array}{c} F_{Jy}l_{1}^{\prime }+K_{2}l_{2}^{\prime }\frac{F_{Jy}+K_{2}l_{2}^{\prime }\theta_{2}}{K_{O2x}+K_{2}}=\left(K_{2}l_{2}^{\prime 2}+K_{O2t}+K_{Ct}+K_{Jt}\right)\theta_{2}, \end{array}$$

(74b) $$\begin{array}{c} F_{Jy}l_{1}^{\prime }\left(K_{O2x}+K_{2}\right)+K_{2}l_{2}^{\prime }\left(F_{Jy}+K_{2}l_{2}^{\prime }\theta_{2}\right)=\left(K_{O2x}+K_{2}\right)\left(K_{2}l_{2}^{\prime 2}+K_{O2t}+K_{Ct}+K_{Jt}\right)\theta_{2}, \end{array}$$

(75) $$\begin{array}{c} \theta_{2}=\frac{l_{1}^{\prime }\left(K_{O2x}+K_{2}\right)+K_{2}l_{2}^{\prime }}{\left(K_{O2x}+K_{2}\right)\left(K_{2}l_{2}^{\prime 2}+K_{O2t}+K_{Ct}+K_{Jt}\right)-K_{2}^{2}l_{2}^{\prime 2}}F_{Jy}, \end{array}$$

(76a) $$\begin{array}{c} \delta_{2}=\frac{F_{Jy}+K_{2}l_{2}^{\prime }l_{1}^{\prime }\left(K_{O2x}+K_{2}\right)+K_{2}l_{2}^{\prime }/\left(K_{O2x}+K_{2}\right)\left(K_{2}l_{2}^{\prime 2}+K_{O2t}+K_{Ct}+K_{Jt}\right)-K_{2}^{2}l_{2}^{\prime 2}F_{Jy}}{K_{O2x}+K_{2}}, \end{array}$$

(76b) $$\begin{array}{c} \delta_{2}=\frac{\left(K_{O2x}+K_{2}\right)\left(K_{2}l_{2}^{\prime 2}+K_{O2t}+K_{Ct}+K_{Jt}\right)-K_{2}^{2}l_{2}^{\prime 2}+K_{2}l_{2}^{\prime }\left[l_{1}^{\prime }\left(K_{O2x}+K_{2}\right)+K_{2}l_{2}^{\prime }\right]}{\left(K_{O2x}+K_{2}\right)\left[\left(K_{O2x}+K_{2}\right)\left(K_{2}l_{2}^{\prime 2}+K_{O2t}+K_{Ct}+K_{Jt}\right)-K_{2}^{2}l_{2}^{\prime 2}\right]}F_{Jy}, \end{array}$$

(76c) $$\begin{array}{c} \delta_{2}=\frac{\left(K_{O2x}+K_{2}\right)\left(K_{2}l_{2}^{\prime 2}+K_{O2t}+K_{Ct}+K_{Jt}\right)-K_{2}^{2}l_{2}^{\prime 2}+K_{2}^{2}l_{2}^{\prime 2}+K_{2}l_{2}^{\prime }l_{1}^{\prime }\left(K_{O2x}+K_{2}\right)}{\left(K_{O2x}+K_{2}\right)\left[\left(K_{O2x}+K_{2}\right)\left(K_{2}l_{2}^{\prime 2}+K_{O2t}+K_{Ct}+K_{Jt}\right)-K_{2}^{2}l_{2}^{\prime 2}\right]}F_{Jy}, \end{array}$$

(76d) $$\begin{array}{c} \delta_{2}=\frac{K_{2}l_{2}^{\prime 2}+K_{O2t}+K_{Ct}+K_{Jt}+K_{2}l_{2}^{\prime }l_{1}^{\prime }}{\left(K_{O2x}+K_{2}\right)\left(K_{2}l_{2}^{\prime 2}+K_{O2t}+K_{Ct}+K_{Jt}\right)-K_{2}^{2}l_{2}^{\prime 2}}F_{Jy}. \end{array}$$

Similarly, the ratio of the amplifier and the input stiffness of beam 2 are determined by

(77) $$\begin{array}{c} \lambda_{2}=\frac{l_{2}^{\prime }\theta_{2}-\delta_{2}}{l_{1}^{\prime }\theta_{2}+\delta_{2}}, \end{array}$$

(78) $$\begin{array}{c} K_{in2}=\frac{F_{Jy}}{l_{1}^{\prime }\theta_{2}+\delta_{2}}. \end{array}$$

Substituting (75) and (76d) into (77) and (78), respectively, the following formulas can be obtained:

(79a) $$\begin{array}{c} \lambda_{2}=\frac{l_{2}^{\prime }\left[l_{1}^{\prime }\left(K_{O2x}+K_{2}\right)+K_{2}l_{2}^{\prime }\right]-\left(K_{2}l_{2}^{\prime 2}+K_{O2t}+K_{Ct}+K_{Jt}+K_{2}l_{2}^{\prime }l_{1}^{\prime }\right)}{l_{1}^{\prime }\left[l_{1}^{\prime }\left(K_{O2x}+K_{2}\right)+K_{2}l_{2}^{\prime }\right]+K_{2}l_{2}^{\prime 2}+K_{O2t}+K_{Ct}+K_{Jt}+K_{2}l_{2}^{\prime }l_{1}^{\prime }}, \end{array}$$

(79b) $$\begin{array}{c} \lambda_{2}=\frac{l_{1}^{\prime }l_{2}^{\prime }\left(K_{O2x}+K_{2}\right)-\left(K_{O2t}+K_{Ct}+K_{Jt}+K_{2}l_{2}^{\prime }l_{1}^{\prime }\right)}{l_{1}^{\prime 2}\left(K_{O2x}+K_{2}\right)+2K_{2}l_{2}^{\prime }l_{1}^{\prime }+K_{2}l_{2}^{\prime 2}+K_{O2t}+K_{Ct}+K_{Jt}}, \end{array}$$

(79c) $$\begin{array}{c} \lambda_{2}=\frac{l_{1}^{\prime }l_{2}^{\prime }K_{O2x}-\left(K_{O2t}+K_{Ct}+K_{Jt}\right)}{l_{1}^{\prime 2}\left(K_{O2x}+K_{2}\right)+2K_{2}l_{2}^{\prime }l_{1}^{\prime }+K_{2}l_{2}^{\prime 2}+K_{O2t}+K_{Ct}+K_{Jt}}, \end{array}$$

(80) $$\begin{array}{c} K_{in2}=\frac{\left(K_{O2x}+K_{2}\right)\left(K_{2}l_{2}^{\prime 2}+K_{O2t}+K_{Ct}+K_{Jt}\right)-K_{2}^{2}l_{2}^{\prime 2}}{l_{1}^{\prime 2}\left(K_{O2x}+K_{2}\right)+2K_{2}l_{2}^{\prime }l_{1}^{\prime }+K_{2}l_{2}^{\prime 2}+K_{O2t}+K_{Ct}+K_{Jt}}, \end{array}$$

(81) $$\begin{array}{c} K_{2}=\frac{K_{Ey}K_{in4}}{K_{Ey}+K_{in4}}. \end{array}$$

Figure 14 presents the force analysis diagram of beam 1.

By a similar consideration to the equilibrium state of the beam 1, the following equations are achieved:

(82) $$\begin{array}{c} F_{O1y}=F_{Ay}+F_{By}, \end{array}$$

(83) $$\begin{array}{c} F_{Ay}l_{1}=F_{By}l_{2}+M_{O1t}+M_{Bt}+M_{At}, \end{array}$$

where *M*_*O1t*_ is the bending moments of the O_1_ point, while M_At_ and M_Bt_ are the bending moments of the A and B points, respectively. The forces acting at points O_1_, A, and B in the direction of *y*-axis are *F*_*O*1*y*_, *F*_*Ay*_, and *F*_*By*_, respectively.

The displacement value at point B is a combination of two displacements including displacement due to hinges at O_1_ rotating around its axis (*l*_*2*_ *θ*_*1*_) and displacement due to drift (*δ*_1_).

Besides, the forces *F*_*O1y*_ and *F*_*By*_ are expressed by

(84) $$\begin{array}{c} F_{By}=K_{1}\left(l_{2}\theta_{1}-\delta_{1}\right), \end{array}$$

(85) $$\begin{array}{c} F_{O1y}=K_{O1x}\delta_{1}, \end{array}$$

(86) $$\begin{array}{c} M_{O1t}=K_{O1t}\theta_{1}, \end{array}$$

(87) $$\begin{array}{c} M_{Bt}=K_{Bt}\theta_{1}, \end{array}$$

(88) $$\begin{array}{c} M_{At}=K_{At}\theta_{1}, \end{array}$$

where *K*_*O1x*_ is the lateral bending stiffness by force *F*_*O1y*_, and *K*_*1*_ is the output stiffness of the B and A points, respectively. *K*_*O1t*_ is the rotational stiffness of the O_1_ point, while *K*_*At*_ and *K*_*Bt*_ are the rotational stiffness of A and B points, respectively.

Similarly, as beam 2, the *θ*_1_ and *δ*_1_ can be derived as

(89) $$\begin{array}{c} \theta_{1}=\frac{l_{1}\left(K_{O1x}+K_{1}\right)+K_{1}l_{2}}{\left(K_{O1x}+K_{1}\right)\left(K_{1}l_{2}^{2}+K_{O1t}+K_{Bt}+K_{At}\right)-K_{1}^{2}l_{2}^{2}}F_{Ay}, \end{array}$$

(90) $$\begin{array}{c} \delta_{1}=\frac{K_{1}l_{1}l_{2}+K_{1}l_{2}^{2}+K_{O1t}+K_{Bt}+K_{At}}{\left(K_{O1x}+K_{1}\right)\left(K_{1}l_{2}^{2}+K_{O1t}+K_{Bt}+K_{At}\right)-K_{1}^{2}l_{2}^{2}}F_{Ay}. \end{array}$$

Regarding beam 1, the ratio of the amplifier and the input stiffness are expressed by

(91) $$\begin{array}{c} \lambda_{1}=\frac{l_{2}\theta_{1}-\delta_{1}}{l_{1}\theta_{1}+\delta_{1}}, \end{array}$$

(92) $$\begin{array}{c} k_{in1}=\frac{F_{Ay}}{l_{1}\theta_{1}+\delta_{1}}. \end{array}$$

Substituting (89) and (90) into (91) and (92), respectively, the results are yielded ass

(93) $$\begin{array}{c} \lambda_{1}=\frac{l_{1}l_{2}K_{O1x}-\left(K_{O1t}+K_{Bt}+K_{At}\right)}{l_{1}^{2}\left(K_{O1x}+K_{1}\right)+2K_{1}l_{1}l_{2}+K_{1}l_{2}^{2}+K_{O1t}+K_{Bt}+K_{At}}, \end{array}$$

(94) $$\begin{array}{c} K_{in1}=\frac{\left(K_{O1x}+K_{1}\right)\left(K_{1}l_{2}^{2}+K_{O1t}+K_{Bt}+K_{At}\right)-K_{1}^{2}l_{2}^{2}}{l_{1}^{2}\left(K_{O1x}+K_{1}\right)+2K_{1}l_{1}l_{2}+K_{1}l_{2}^{2}+K_{O1t}+K_{Bt}+K_{At}}, \end{array}$$

(95) $$\begin{array}{c} K_{1}=K_{12}+K_{13}, \end{array}$$

(96) $$\begin{array}{c} K_{12}=\frac{K_{y12}K_{in2}}{K_{y12}+K_{in2}}, \end{array}$$

(97) $$\begin{array}{c} K_{y12}=\frac{K_{\theta yMy}^{e}}{2}+\frac{2K_{\theta yMy}^{e}}{5}=\frac{9K_{\theta yMy}^{e}}{10}, \end{array}$$

(98) $$\begin{array}{c} K_{13}=\frac{K_{y13}K_{in3}}{K_{y13}+K_{in3}}, \end{array}$$

(99) $$\begin{array}{c} K_{y13}=\frac{2K_{\theta yMy}^{e}}{5}, \end{array}$$

where *K*_*1*_ is the output stiffness that combines A and B points, *K*_12_ is the output stiffness that combines beam 1 and beam 2, and *K*_13_ is the output stiffness that combines beam 1 and beam 3. In addition, the intermediary stiffness between beam 1 and beam 2, and the intermediary stiffness between beam 1 and beam 3 are *K*_*y*12_ and *K*_*y*13_, respectively.

(100) $$\begin{array}{c} K_{O1t}=K_{O2t}=K_{O4t}=K_{At}=K_{\theta zMz}^{e}, \end{array}$$

(101) $$\begin{array}{c} K_{Bt}=K_{Ct}=K_{Dt}=K_{It}=K_{O3t}=K_{Et}=\frac{K_{\theta zMz}^{e}}{2}, \end{array}$$

(102) $$\begin{array}{c} K_{Ht}=K_{Jt}=K_{Mt}=K_{Nt}=\frac{2K_{\theta zMz}^{e}}{5}, \end{array}$$

(103) $$\begin{array}{c} K_{Gt}=K_{\theta zMz}^{c}, \end{array}$$

(104) $$\begin{array}{c} K_{O3y}=K_{Ey}=K_{Cy}=\frac{K_{\theta yMy}^{e}}{2}, \end{array}$$

(105) $$\begin{array}{c} K_{O1x}=K_{O2x}=K_{O4x}=K_{\delta xFx}^{e}, \end{array}$$

(106) $$\begin{array}{c} K_{O3x}=\frac{K_{\delta xFx}^{e}}{2}, \end{array}$$

(107) $$\begin{array}{c} K_{b2}=K_{\theta zMz}^{l7}=\frac{Eba^{3}}{12l_{7}}, \end{array}$$

(108) $$\begin{array}{c} K_{\theta yMy}^{l7}=\frac{Eba}{l_{7}}, \end{array}$$

(109) $$\begin{array}{c} K_{b1}=K_{\theta zMz}^{l8}=\frac{Eba^{3}}{12l_{8}}, \end{array}$$

(110) $$\begin{array}{c} K_{\theta zMz}^{l9}=\frac{Eba^{3}}{12l_{9}}, \end{array}$$

(111) $$\begin{array}{c} K_{b3}=\frac{2K_{\theta zMz}^{l9}K_{\theta zMz}^{c}}{K_{\theta zMz}^{c}+4K_{\theta zMz}^{l9}}, \end{array}$$

where *K*_*θzMz*_^*l*7^, *K*_*θzMz*_^*l*8^, and *K*_*θzMz*_^*l*9^ are the stiffnesses which are created via the torque of the leaf hinges *l*_*7*_, *l*_*8*_, and *l*_*9*_, respectively. In addition, *K*_*b*1_, *K*_*b*2_, and *K*_*b*3_ are stiffness conversion of *K*_*θzMz*_^*l*7^, *K*_*θzMz*_^*l*8^, and *K*_*θzMz*_^*l*9^, the purpose of this action is making the equations simpler. Finally, the linear stiffness is created via the axial force of the leaf hinge *l*_7_, also display by value *K*_*θyMy*_^*l*7^.

Finally, the total ratio of two-stage lever amplifier is computed by

(112) $$\begin{array}{c} A=\lambda_{4}\left(\lambda_{1}+\lambda_{3}+1\right)+\lambda_{3}+1. \end{array}$$

It is found that *F*_*in*_ *=* *2F*_*Ay*_, and considering the symmetric amplifier, the input stiffness is expressed by

(113) $$\begin{array}{c} K_{in}=\frac{F_{in}}{l_{1}\theta_{1}+\delta_{1}}=\frac{2F_{Ay}}{l_{1}\theta_{1}+\delta_{1}}=2K_{in1}. \end{array}$$

**Figure 15:**
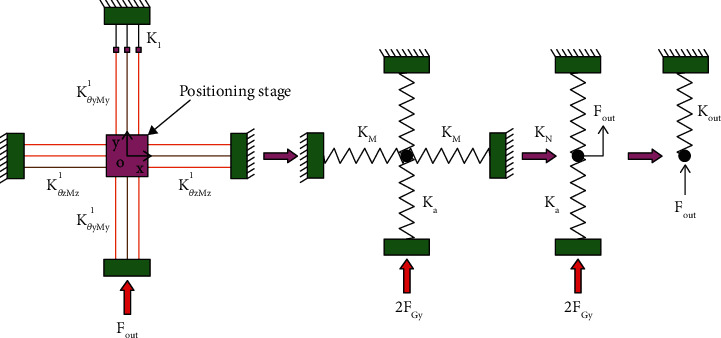
The stiffness of the output mechanism.

**Figure 16:**
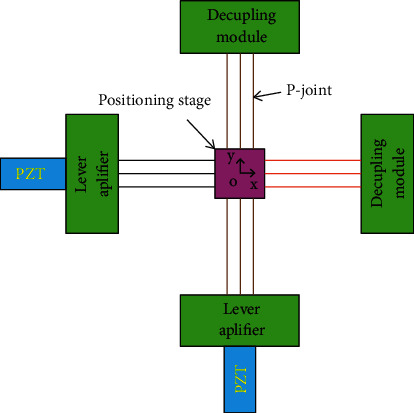
Simplified principle diagram of the 2-dof stage.

A simplified stiffness diagram of the stage is given in Figure 15 when the platform is driven in the direction of *y*-axis.  Figure 16 shows a simplified principle diagram of the 2-dof stage.

Let *d*_*out*_ be the output displacement of the stage and *d*_*in*_ be the input displacement in the vertical direction. Besides, the rotational angles of the prismatic beams are symbolled as *ω*_*b*1_, *ω*_*b*2_, *ω*_*b*3_, *α*_1_, *β*_1_, *γ*_1_, and *ε*_1_, which can be calculated by

(114) $$\begin{array}{c} \omega_{b1}=\frac{d_{out}}{l_{8}}, \end{array}$$

(115) $$\begin{array}{c} \omega_{b2}=\frac{d_{out}}{l_{7}}, \end{array}$$

(116) $$\begin{array}{c} \omega_{b3}=\frac{d_{out}}{l_{9}}, \end{array}$$

(117) $$\begin{array}{c} \alpha_{1}=\frac{d_{out}}{l_{5}}, \end{array}$$

(118) $$\begin{array}{c} \beta_{1}=\frac{d_{out}}{\left(l_{3}+l_{4}\right)}\frac{l_{6}}{l_{5}}, \end{array}$$

(119) $$\begin{array}{c} \gamma_{1}=\frac{d_{out}}{l_{2}{\;}^{,}}\frac{l_{6}}{l_{5}}, \end{array}$$

(120) $$\begin{array}{c} \varepsilon_{1}=\frac{d_{out}}{l_{2}}\frac{l_{6}}{l_{5}}. \end{array}$$

The potential energy inside the flexure hinges is expressed by

(121a) $$\begin{array}{c} E_{p}=\frac{1}{2}\left[\sum_{i=A}^{B}K_{i}\varepsilon^{2}{\;}_{1}+\sum_{i=J}^{D}K_{i}\gamma^{2}{\;}_{1}+\sum_{i=O4}^{I}K_{i}\beta^{2}{\;}_{1}+K_{G}\alpha^{2}{\;}_{1}\right]+\frac{1}{2}\sum_{j=b1}^{b3}K_{j}\omega^{2}{\;}_{j}, \end{array}$$

(121b) $$\begin{array}{c} E_{p}=\frac{1}{2}\left[\;\right)K_{At}\varepsilon_{1}{\;}^{2}+K_{O1t}\varepsilon_{1}{\;}^{2}+K_{Bt}\varepsilon_{1}{\;}^{2}+K_{Jt}\gamma_{1}{\;}^{2}+K_{O2t}\gamma_{1}{\;}^{2}+K_{Dt}\gamma_{1}{\;}^{2} \end{array}$$

Substituting (114)–(120) into (121b), the stiffness *K*_*N*_ can be achieved by

(122) $$\begin{array}{c} K_{N}=2\left[\begin{array}{c} {\left(\frac{l_{6}}{l_{2}l_{5}}\right)}^{2}\left(K_{At}+K_{O1t}+K_{Bt}\right)+{\left(\frac{l_{6}}{l^{\prime }{\;}_{2}l_{5}}\right)}^{2}\left(K_{Jt}+K_{O2t}+K_{Dt}\right)\\ +{\left(\frac{l_{6}}{\left(l_{4}+l_{3}\right)l_{5}}\right)}^{2}\left(K_{O4t}+K_{Ht}+K_{It}\right)+{\left(\frac{1}{l_{5}}\right)}^{2}K_{Gt} \end{array}\right]\\ +\left[4{\left(\frac{1}{l_{8}}\right)}^{2}K_{b1}+6{\left(\frac{1}{l_{7}}\right)}^{2}K_{b2}+2{\left(\frac{1}{l_{9}}\right)}^{2}K_{b3}\right]. \end{array}$$

Next, the output stiffness *K*_*out*_ and *K*_*Gl*_ of the stage are determined by

(123) $$\begin{array}{c} K_{out}=\frac{K_{N}K_{a}}{K_{N}+K_{a}}, \end{array}$$

in which *K*_*a*_ as shown in Figure 15 and calculated by

(124) $$\begin{array}{c} K_{a}=\frac{3K_{\theta yMy}^{l7}}{2}, \end{array}$$

(125) $$\begin{array}{c} K_{Gl}=0.5K_{out}, \end{array}$$

where *K*_*N*_ is the combines stiffness of the micromanipulator in one direction, *K*_*a*_ is the stiffness of the beam *K*_*θyMy*_^*l*7^, and *K*_*out*_ is the output stiffness of the micromanipulator.

For a simple calculation, the geometric parameters and of the AL7075-T6 properties of the stage are provided in Table 3.

#### 4.1.2. Modeling of Working Workspace

Assuming that the A is the ratio of amplifier and *S* is the stroke of the PZT, the workspace of the stage is calculated as AS x AS. I axial tension and shearing effects are ignored. There is only the bending stress is considered. To sum up, the stress (*σ*_*r*_) is computed by

(126) $$\begin{array}{c} Max\left(\sigma_{r}\right)=\frac{\sigma_{y}}{s}, \end{array}$$

where *σ*_*y*_ is the yield strength of the material and *s* is the safety factor.

The maximal stress *σ*_*r*_^*Max*^ is computed as

(127) $$\begin{array}{c} \sigma_{r}^{Max}=\frac{4Er^{2}K_{c}}{f\left(\beta \right)t^{2}}\theta_{Max}, \end{array}$$

where *k*_*c*_ is concentration factor and *f*(*β*) is the compliance factor [22].

(128) $$\begin{array}{c} \beta =\frac{t}{2r}, \end{array}$$

(129) $$\begin{array}{c} k_{c}={\left(1+\beta \right)}^{\frac{9}{20}}, \end{array}$$

(130) $$\begin{array}{c} f\left(\beta \right)=\frac{1}{2\beta +\beta^{2}}\left(\frac{3+4\beta +2\beta^{2}}{\left(1+\beta \right)\left(2\beta +\beta^{2}\right)}+\frac{6\left(1+\beta \right)}{{\left(\sqrt{2\beta +\beta^{2}}\right)}^{3}}\tan^{-1}\sqrt{\frac{2+\beta }{\beta }}\right), \end{array}$$

where *β* is the dimensionless geometry factor.

Let *δ*_*in*_^*Max*^ is the maximal input displacement and *δ*_*in*_^*Max*^ is the output displacement of stage. The maximal deformation angle (*θ*_max_) is computed by

(131) $$\begin{array}{c} \theta_{Max}=\frac{A\delta_{in}^{Max}}{l_{5}}+\frac{\lambda_{3}\delta_{in}^{Max}}{l_{3}+l_{4}}. \end{array}$$

Substituting (126) and (131) into (127), the results are yielded as

(132) $$\begin{array}{c} \frac{\sigma_{y}}{s}\geq \frac{4Er^{2}K_{c}}{f\left(\beta \right)t^{2}}\left(\frac{A\delta_{in}^{Max}}{l_{5}}+\frac{\lambda_{3}\delta_{in}^{Max}}{l_{3}+l_{4}}\right), \end{array}$$

(133) $$\begin{array}{c} \delta_{in}^{Max}\leq \frac{l_{5}\left(l_{3}+l_{4}\right)f\left(\beta \right)t^{2}\sigma_{y}}{4Er^{2}K_{c}\;s\left(A\left(l_{3}+l_{4}\right)+\lambda_{3}l_{5}\right)}. \end{array}$$

By using the values in Table 2, the (133) and let *s* of 1.8, the maximal input displacement is expressed by

(134) $$\begin{array}{c} \delta_{in}^{Max}\leq 44\text{.}6286\;\mu m, \end{array}$$

To sum up, the output displacement of the stage may achieve up to 1363.8 *μ*m. Therefore, the workspace of the 2-dof stage is 1363.8 *μ*m × 1363.8 *μ*m.

#### 4.1.3. Modeling of Dynamics

**Figure 17:**
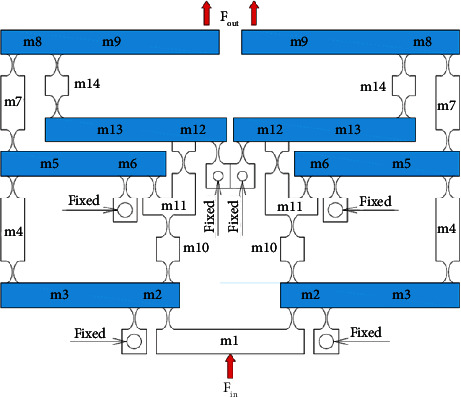
Symmetrical structure with two 2-stage amplifiers.

The natural frequencies can be achieved by Lagrange's method by calculating the kinetic energy (*T*) and potential energy (*V*). Figures 12 and 17 show the position of masses *m* in the proposed design. Based on the position and motion trajectory of the components, a motion classification step will be performed to assist the calculation of the natural frequency of the design.

The hinges *m*_*2*_, *m*_*3*_, *m*_*5*_, *m*_*6*_, *m*_*8*_, *m*_*9*_, *m*_*12*_, *m*_*13*_, *m*_*b1*_, *m*_*b2*_, *m*_*b3*_ represent the rotational and translational motions. The hinges *m*_*0*_, *m*_*1*_, *m*_*4*_, *m*_*7*_, *m*_*10*_, *m*_*11*_, *m*_*14*_, *m*_*15*_, *m*_*16*_ note the translations. The kinetic energy of the whole stage is computed as

(135) $$\begin{array}{c} T=T_{\eta_{1}}+T_{\eta_{2}}, \end{array}$$

(136) $$\begin{array}{c} T_{\eta_{1}}=T_{\eta_{2}}=\sum_{i=m_{2}}^{m_{16}}T_{i}+\sum_{j=m_{b1}}^{m_{b3}}T_{j}, \end{array}$$

(137) $$\begin{array}{c} T_{m_{0}}=\frac{1}{2}m_{0}{\left(vA\right)}^{2}=\frac{1}{2}m_{0}{\left(\eta_{1}^{.}A\right)}^{2}=\frac{m_{0}A^{2}}{2}\eta_{1}^{.2}, \end{array}$$

(138) $$\begin{array}{c} T_{m_{1}}=\frac{m_{1}}{2}\eta_{1}^{.2}, \end{array}$$

(139) $$\begin{array}{c} T_{m_{4}}=\frac{1}{2}m_{4}{\left(\frac{v\lambda_{1}}{2}\right)}^{2}2=\frac{m_{4}\lambda_{1}^{2}}{4}\eta_{1}^{.2}, \end{array}$$

(140) $$\begin{array}{c} T_{m_{7}}=\frac{m_{7}\lambda_{2}^{2}}{4}\eta_{1}^{.2}, \end{array}$$

(141) $$\begin{array}{c} T_{m_{10}}=\frac{m_{10}}{4}\eta_{1}^{.2}, \end{array}$$

(142) $$\begin{array}{c} T_{m_{11}}=\frac{m_{11}}{4}\eta_{1}^{.2}, \end{array}$$

(143) $$\begin{array}{c} T_{m_{15}}=\frac{m_{15}A^{2}}{2}\eta_{1}^{.2}, \end{array}$$

(144) $$\begin{array}{c} T_{m_{16}}=\frac{m_{16}A^{2}}{2}\eta_{1}^{.2}, \end{array}$$

(145) $$\begin{array}{c} T_{m_{2}}=\frac{1}{2}m_{2}{\left(\frac{v}{2}\right)}^{2}2+\frac{1}{2}\frac{m_{2}l_{1}^{2}}{3}{\left(\frac{v}{l_{1}}\right)}^{2}2=\frac{7m_{2}}{12}v^{2}=\frac{7m_{2}}{12}\eta_{1}^{.2}, \end{array}$$

(146) $$\begin{array}{c} T_{m_{3}}=\frac{7m_{3}\lambda_{1}^{2}}{12}\eta_{1}^{.2}, \end{array}$$

(147) $$\begin{array}{c} T_{m_{5}}=\frac{7m_{5}\lambda_{2}^{2}}{12}\eta_{1}^{.2}, \end{array}$$

(148) $$\begin{array}{c} T_{m_{6}}=\frac{7m_{6}}{12}\eta_{1}^{.2}, \end{array}$$

(149) $$\begin{array}{c} T_{m_{8}}=\frac{7m_{8}A^{2}}{12}\eta_{1}^{.2}, \end{array}$$

(150) $$\begin{array}{c} T_{m_{9}}=\frac{7m_{9}A^{2}}{12}\eta_{1}^{.2}, \end{array}$$

(151) $$\begin{array}{c} T_{m_{12}}=\frac{7m_{12}}{12}\eta_{1}^{.2}, \end{array}$$

(152) $$\begin{array}{c} T_{m_{13}}=\frac{7m_{13}\lambda_{3}^{2}}{12}\eta_{1}^{.2}, \end{array}$$

(153) $$\begin{array}{c} T_{m_{b1}}=\frac{1}{2}m_{b1}{\left(vA\right)}^{2}4+\frac{1}{2}\frac{m_{b1}l_{8}^{2}}{3}{\left(\frac{vA}{l_{8}}\right)}^{2}4=\frac{8m_{b1}A^{2}}{3}v^{2}=\frac{8m_{b1}A^{2}}{3}\eta_{1}^{.2}, \end{array}$$

(154) $$\begin{array}{c} T_{m_{b2}}=\frac{1}{2}m_{b2}{\left(vA\right)}^{2}6+\frac{1}{2}\frac{m_{b2}l_{7}^{2}}{3}{\left(\frac{vA}{l_{7}}\right)}^{2}6=4A^{2}m_{b2}\eta_{1}^{.2}, \end{array}$$

(155) $$\begin{array}{c} T_{m_{b3}}=\frac{1}{2}m_{b3}{\left(vA\right)}^{2}2+\frac{1}{2}\frac{m_{b3}l_{9}^{2}}{3}{\left(\frac{vA}{l_{9}}\right)}^{2}2=\frac{4A^{2}m_{b3}}{3}\eta_{1}^{.2}. \end{array}$$

Potential energy:

(156) $$\begin{array}{c} V=\frac{1}{2}k_{1}\eta_{1}^{2}+\frac{1}{2}k_{2}\eta_{2}^{2}. \end{array}$$

Lagrange's equation: Lagrangian mechanics defines a mechanical system to be a pair (*M*, *L*) of a configuration space *M* and a smooth function *L* = *L*(*q*, *v*,*t*) called Lagrangian.

(157) $$\begin{array}{c} L=T-V,\\ \frac{d}{d_{t}}\frac{\partial T}{\partial \mu_{i}^{*}}-\frac{\partial T}{\partial \mu_{i}}+\frac{\partial V}{\partial \mu_{i}}=F_{i},\\ Md_{i}^{**}+Kd_{i}=F_{i}, \end{array}$$

where *i* = 1; 2 is the free vibration of horizontal and vertical directions of the stage. *F*_*i*_ is a nonconservative generalized force corresponding to the coordinate. Let the equivalent mass *M*=*diag*{*M*} and the stiffness *K*=*diag*{*k*}, a conservative system is expressed by

(158) $$\begin{array}{c} M_{\eta^{**}}+K_{\eta }=0, \end{array}$$

(159) $$\begin{array}{c} \frac{d}{d_{t}}\frac{\partial T}{\partial \mu^{*}{\;}_{i}}-\frac{\partial T}{\partial \mu_{i}}+\frac{\partial V}{\partial \mu_{i}}=0. \end{array}$$

Substituting (136)–(155) into (135), the results are yielded as

(160) $$\begin{array}{c} T=\left[\frac{m_{0}A^{2}}{2}\right)+\frac{m_{1}}{2}+\frac{7m_{2}}{12}+\frac{7m_{3}\lambda_{1}^{2}}{12}+\frac{m_{4}\lambda_{1}^{2}}{4}+\frac{7m_{5}\lambda_{2}^{2}}{12}+\frac{7m_{6}}{12}+\frac{m_{7}\lambda_{2}^{2}}{4}+\frac{7m_{8}A^{2}}{12}\\ +\frac{7m_{9}A^{2}}{12}+\frac{m_{10}}{4}+\frac{m_{11}}{4}+\frac{7m_{12}}{12}+\frac{7m_{13}\lambda_{3}^{2}}{12}+\frac{m_{14}\lambda_{3}^{2}}{4}+\frac{m_{15}A^{2}}{2}+\frac{m_{16}A^{2}}{2}+\frac{8m_{b1}A^{2}}{3}\\ +4A^{2}m_{b2}+\left(\frac{4A^{2}m_{b3}}{3}\right]\left[\eta^{*2}{\;}_{1}\eta^{*2}{\;}_{2}\right], \end{array}$$

(161) $$\begin{array}{c} \frac{\partial T}{\partial \mu^{*}{\;}_{i}}=\left[m_{0}A^{2}\right)+m_{1}+\frac{7m_{2}}{6}+\frac{7m_{3}\lambda_{1}^{2}}{6}+\frac{m_{4}\lambda_{1}^{2}}{2}+\frac{7m_{5}\lambda_{2}^{2}}{6}+\frac{7m_{6}}{6}+\frac{m_{7}\lambda_{2}^{2}}{2}+\frac{7m_{8}A^{2}}{6}\\ \;+\frac{7m_{9}A^{2}}{6}+\frac{m_{10}}{2}+\frac{m_{11}}{2}+\frac{7m_{12}}{6}+\frac{7m_{13}\lambda_{3}^{2}}{6}+\frac{m_{14}\lambda_{3}^{2}}{2}+m_{15}A^{2}+m_{16}A^{2}+\frac{16m_{b1}A^{2}}{3}\\ \;+8A^{2}m_{b2}+\left(\frac{8A^{2}m_{b3}}{3}\right]\left[\eta^{*}{\;}_{1}\eta^{*}{\;}_{2}\right],\\ \;+\frac{7m_{9}A^{2}}{6}+\frac{m_{10}}{2}+\frac{m_{11}}{2}+\frac{7m_{12}}{6}+\frac{7m_{13}\lambda_{3}^{2}}{6}+\frac{m_{14}\lambda_{3}^{2}}{2}+m_{15}A^{2}+m_{16}A^{2}+\frac{16m_{b1}A^{2}}{3}\\ \;+8A^{2}m_{b2}+\left(\frac{8A^{2}m_{b3}}{3}\right]\left[\eta^{**}{\;}_{1}\eta^{*\&lowast;}{\;}_{2}\right], \end{array}$$

(162) $$\begin{array}{c} \frac{d}{d_{t}}\frac{\partial T}{\partial \mu^{*}{\;}_{i}}=\left[m_{0}A^{2}\right)+m_{1}+\frac{7m_{2}}{6}+\frac{7m_{3}\lambda_{1}^{2}}{6}+\frac{m_{4}\lambda_{1}^{2}}{2}+\frac{7m_{5}\lambda_{2}^{2}}{6}+\frac{7m_{6}}{6}+\frac{m_{7}\lambda_{2}^{2}}{2}+\frac{7m_{8}A^{2}}{6}\\ \;+\frac{7m_{9}A^{2}}{6}+\frac{m_{10}}{2}+\frac{m_{11}}{2}+\frac{7m_{12}}{6}+\frac{7m_{13}\lambda_{3}^{2}}{6}+\frac{m_{14}\lambda_{3}^{2}}{2}+m_{15}A^{2}+m_{16}A^{2}+\frac{16m_{b1}A^{2}}{3}\\ \;+8A^{2}m_{b2}+\left(\frac{8A^{2}m_{b3}}{3}\right]\left[\eta^{**}{\;}_{1}\eta^{*\&lowast;}{\;}_{2}\right], \end{array}$$

(163) $$\begin{array}{c} \frac{\partial T}{\partial \mu_{i}}=0, \end{array}$$

(164) $$\begin{array}{c} \frac{\partial V}{\partial \mu_{i}}=\left[k_{1}+k_{2}\right]\left[\eta_{1}\eta_{2}\right]. \end{array}$$

Substituting (162)–(164) into (159), values *M* and *K* can be expressed by the following formula:

(165) $$\begin{array}{c} M=m_{0}A^{2}+m_{1}+\frac{7m_{2}}{6}+\frac{7m_{3}\lambda_{1}^{2}}{6}+\frac{m_{4}\lambda_{1}^{2}}{2}+\frac{7m_{5}\lambda_{2}^{2}}{6}+\frac{7m_{6}}{6}+\frac{m_{7}\lambda_{2}^{2}}{2}\\ \;+m_{16}A^{2}+\frac{16m_{b1}A^{2}}{3}+8A^{2}m_{b2}+\frac{8A^{2}m_{b3}}{3}, \end{array}$$

(166) $$\begin{array}{c} K=K_{in}=2K_{in1}. \end{array}$$

Solving (158), the natural frequency (*f*) of the stage can be obtained as

(167) $$\begin{array}{c} f=\frac{1}{2\pi }{\left(\frac{K}{M}\right)}^{0.5}. \end{array}$$

### 4.2. Evaluation and Verifications of Mathematical Models

Table 4 shows that an error between the theoretical result and FEA result is about 5.426%. It means that the proposed methodology based on the kinetostatic-based methods (i.e., defomable mechanic theory, elastic beam theory, and Lagrange method) is reliable and effective in modeling the statics and dynamics of the 2-dof stage.

### 4.3. Structural Optimization

#### 4.3.1. Optimization Problem Description

To avoid the resonance phenomena between the motors, PZT actuators, and the compliant 2-dof stage, the first natural frequency modes are either as small as possible or as large as possible. In order to increase the rapid responsiveness of the positioners, the first natural frequency should be chosen as large as possible. In addition, the angular frequency is proportional to the natural frequency of the compliant stage. Therefore, the first natural frequency is proposed to maximize in order to increase the response speed as well as avoid the resonance phenomena of the 2-dof stage. In this research, the optimization problem is aimed to maximize the resonant frequency, which is briefly expressed as follows.

Find design vector: **x**=[*x*_1_, *x*_2_, *x*_3_, *x*_4_, *x*_5_]Maximize

(168) $$\begin{array}{c} \;f\left(x\right), \end{array}$$

S.t:

(169) $$\begin{array}{c} \text{f}\left(\text{x}\right)\;>\;100\text{ Hz}, \end{array}$$

Design variables (unit: mm):

(170) $$\begin{array}{c} \left\{\begin{array}{c} 0.7\leq x_{1}\leq 0.9\\ 0.6\leq x_{2}\leq 0.8\\ 0.6\leq x_{3}\leq 0.75\\ 0.6\leq x_{4}\leq 0.7\\ 0.6\leq x_{5}\leq 0.7 \end{array}\right), \end{array}$$

where *f*(*x*) denotes the resonant frequency. In addition, *x*_*1*_, *x*_*2*_, *x*_*3*_, *x*_*4*_, and *x*_*5*_ are the dimensions of *A*, *B*, *C*, *D*, and *E*, respectively.

#### 4.3.2. Optimal Results

Based on (1)–(170), MATLAB R2017b was employed for developing the combination approach of the kinetostatic analysis-based method and the neural network algorithm. As a result, the optimal parameters of the stage were found at *A* = 0.9 mm, *B* = 0.8 mm, *C* = 0.7 mm, *D* = 0.7 mm, and *E* = 0.7 mm, and the first natural frequency was 112.0995 Hz.

### 4.4. Verification and Comparisons

**Figure 18:**
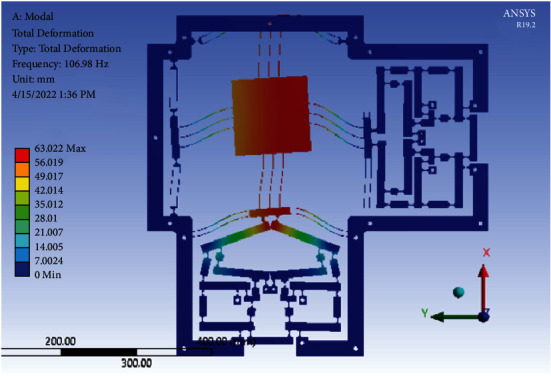
The first mode shape analysis of the resonant natural frequency of the optimal 2-dof stage.

By using the optimal parameters, a 3D stage is created. The FEA result in ANSYS software showed that the first natural frequency was 106.98 Hz. Compared with the initial design, the frequency of the 2-dof stage is improved by up to 21.69%, as given in Table 5. In addition, the error among the optimal value and FEA value is 4.785%, as given in Table 6. Besides, the resonant frequency values of the six modes from mode 1 to mode 6 are 106.98 Hz, 121.75 Hz, 328.69 Hz, 335.46 Hz, 602.42 Hz, and 603.88 Hz, respectively.  Figure 18 illustrates the first mode shape analysis of the resonant natural frequency. So as to prevent the damage of the optimal positioner due to resonances, the positioner should avoid the abovementioned resonant frequencies during operation.

Compared with the previous designs of the 2-dof stage, the present design proposed a higher frequency, as given in Table 7.

**Figure 19:**
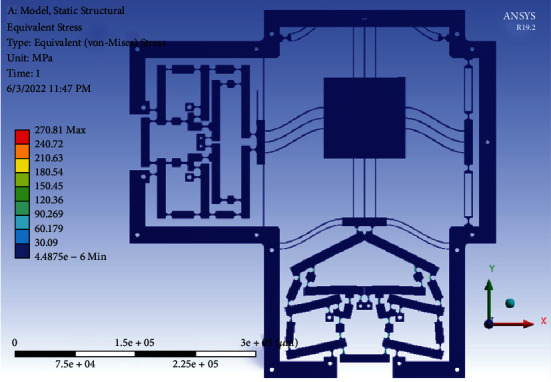
The equivalent stress of the optimal 2-dof stage.

**Figure 20:**
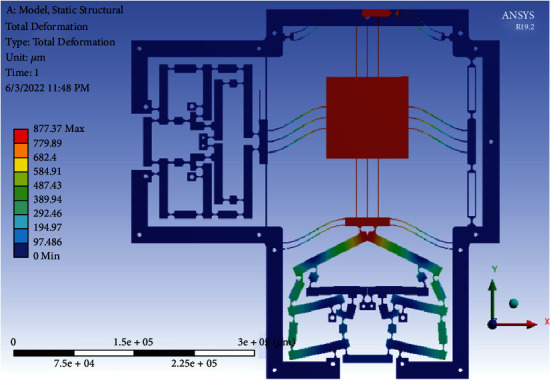
The total deformation of the optimal 2-dof stage.

In addition, in order to evaluate the material strength of the optimal stage, the FEA result in ANSYS software showed that the equivalent stress was 270.81 MPa, as illustrated in Figure 19. The output total deformation was 877.37 *μ*m, as depicted in Figure 20. Besides, the displacement amplification ratio was 7.79, the output force was 3480.4 N, and the parasitic motion error, so-called as the cross-axis coupling ratio between the two axes, was around 0.02 %. Specifically, the achieved working stroke of the XY stage was 787.63 *μ*m × 794.5 *μ*m. The safety factor is found about 1.86 (i.e., the resulting stress is much smaller than the yield strength of used material). The achieved optimal results satisfied the desirable technical specifications of the XY stage.

## 5. Conclusions

This study presented a new modeling and dimensional optimization synthesis of the 2-dof stage. The developed 2-dof stage would be applied for positioning a biomaterial sample in in situ nanoindentation. The proposed effective methodology was proposed according to the kinetostatic analysis-based calculation method, the Lagrange approach, and the neural network algorithm. First of all, the 2-dof stage was designed using a hybrid integration of an eight-lever displacement amplifier-integrated elliptical hinges and a symmetric parallelogram. Then, a chain of mathematical equations of the 2-dof stage were formulated using a kinetostatic analysis-based method in terms of the ratio of displacement amplification and the input stiffness. Subsequently, the Lagrange method was employed to form the dynamic equation of the 2-dof stage. Finally, the neural network algorithm was adopted to maximize the natural first frequency of the proposed stage. The optimal results indicated that the frequency of the stage achieved a high value of 112.0995 Hz. Besides, the mathematical models were relatively close to the simulation verifications. The designed stage has a faster response than that from a few previous designs.

In the future study, a prototype of the 2-dof stage will be manufactured, and the analytical results will be verified by the experiments. In addition, the closed loop system will be utilized for verifying the analytical results as well as enhancing position accuracy of the 2-dof stage.

## Figures and Tables

**Table 1:** Main geometric parameters of the proposed 2-dof stage.

Factor	Value	Factor	Value	Unit
a	451	k	100	mm
b	451	m	100	mm
c	248	p	54	mm
d	248	A	0.9 ≤ A ≤ 1.1	mm
e	20	B	0.7 ≤ B ≤ 0.9	mm
f	248	C	0.6 ≤ C ≤ 0.8	mm
g	248	D	0.6 ≤ D ≤ 0.8	mm
h	54	E	0.6 ≤ E ≤ 0.7	mm

**Table 2:** The desirable technical specifications of the XY stage.

Technical specifications	Desirable results
Workspace size	451 × 451 (mm)
Desirable working stroke	770 × 770 (*μ*m)
Desirable resonant frequency	100 (Hz)
Desirable displacement amplification ratio	7
Parasitic motion error/cross-axis coupling ratio	0.04 (%)
Safety factor	1.7
Range of the maximum load	3000 N

**Table 3:** Geometric parameters and AL7075-T6 for the proposed stage.

*l*_*1*_	*l*_*2*_	*l'*_*1*_	*l'*_*2*_	*l*_*3*_	*l*_*4*_	*l*_*5*_	*l*_*6*_	*l*_*7*_	*l*_*8*_	*l*_*9*_
13.9	49.7	12	46	14	52	61.8	18	73	76.9	65.9
*r*	*t* (circular)	a (*l*_*8*_, *l*_*9*_)	a (*l*_*7*_)	*ax*	*ay*	*t* (elliptical)	*b*	*l*	*E* (GPa)	*σ *_*r*_ (MPa)
4.5	0.6	0.6	0.8	4.5	2.5	0.65	16	9	71.7	503

**Table 4:** Error between the theory and simulation result.

Response	Analytical result	FEA result	Error (%)
*f* (Hz)	92.1219	87.381	5.426

**Table 5:** Improvement between optimal result and initial design result.

Response	Optimal result	Initial design result	Improvement (%)
*f* (Hz)	112.0995	92.1219	21.69

**Table 6:** Error between optimal result and FEA result.

Response	Optimal result	FEA result	Error (%)
*f* (Hz)	112.0995	106.98	4.785

**Table 7:** Comparison of the proposed 2-dof stage with the previous designs.

Design of the 2-dof stage	Dimension	Frequency (Hz)
Zhu et al. [23]	NA	59.3
Lee et al. [24]	NA	80
Present design	451 mm × 451 mm × 16 mm	**112.0995**

## Data Availability

The data used to support the findings of this study are included within the article.

## Abbreviations

2-dof: Two degrees of freedom
MDLA: Modified displacement lever amplifier
LAM 1: Lever amplification mechanism of floor 1
LAM 2: Lever amplification mechanism of floor 2
LAM 3: Lever amplification mechanism of floor 3
*l*_*1*_: Length of segment AO_1_
*l*_*2*_: Length of segment O_1_B
*l*_*1*_ *'*: Length of segment JO_2_
*l*_*2*_ *'*: Length of segment O_2_C
*l*_*3*_: Length of segment O_4_ H
*l*_*4*_: Length of segment HI
*l*_*5*_: Length of segment EO_3_
*l*_*6*_: Length of segment O_3_G
*A*: The amplification ratio
*δ*_*in*_: Input displacement
*δ*_*out*_: Output displacement
*K*_*θzMz*_^*c*^: The torsion stiffness made via the torque of a flexure circular notched hinge
*K*_*θyMy*_^*c*^: The linear stiffness created via the axial force of a flexure circular notched hinge
*K*_*δxFx*_^*c*^: The bending stiffness generated via lateral force of a flexure circular notched hinge
*K*_*θzMz*_^*l*^: The torsion stiffness made via the torque of the leaf hinge
*K*_*θyMy*_^*l*^: The linear stiffness created via the axial force of the leaf hinge
Δ*θ*: The variable angle created via the torque of the leaf hinge
*M*_*θzMz*_^*l*^: The bending moments of the leaf hinge
*K* ^*e*^ _*θzMz*_: The torsion stiffness made via the torque of a flexure elliptical notched hinge
*K*_*θyMy*_^*e*^: The linear stiffness created via the axial force of a flexure elliptical notched hinge
*K*_*δ*_*X*_ *F*_*X*__^*e*^: The bending stiffness generated via lateral force of a flexure elliptical notched hinge
*E*: The elastic modulus of the manufacturing material
*b*: The thickness of the flexure hinge
*r*: The radius of the circular notched hinge
*t*: The smallest width of the circular/elliptical hinge
*l*: The length of the flexure hinge
*a*: The width of the flexure hinge
*a*_*x*_: The major axis of the elliptical flexure hinge
*a*_*y*_: The minor axis of the elliptical flexure hinge
*β*_*X*_: The dimensionless factor representing the hinges' geometry *a*_*x*_
*β*_*y*_: The dimensionless factor representing the hinges' geometry *a*_*y*_
∈: The multiplication factor of the ratio of the major to minor axes
*f*(*β*_*y*_): The dimensionless compliance factor according to *β*_*y*_
*f*(*β*_*x*_): The dimensionless compliance factor according to *β*_*X*_
*K*_*in*_: The input stiffness of the micromanipulator
*F*_*in*_: The input force of the micromanipulator
*F*_*O*3*y*_: The force acting at point O_3_ in the direction of *y*-axis
*F*_*Ey*_: The force acting at point *E* in the direction of *y*-axis
*F*_*Gy*_: The force acting at point G in the direction of *y*-axis
*M*_*O*3*t*_: The bending moments at points O_3_
*M*_*Gt*_: The bending moments at points G
*M*_*Et*_: The bending moments at points E
*K*_*O*3*x*_: The lateral bending stiffness by the force *F*_*O3y*_
*δ*_4_: The displacement due to drift of beam 4
*K*_*Gy*_: The G point's output stiffness of the MDLA
*θ*_4_: The angle variable is created via the torque of beam 4
*K*_*O*3*t*_: The rotational stiffness of points O_3_
*K*_*Gt*_: The rotational stiffness of points G
*K*_*Et*_: The rotational stiffness of points E
*λ*_4_: The amplification ratio amplifier of beam 4
*K*_*in*4_: The input stiffness of beam 4
*F*_*Hy*_: The force acting at point H in the direction of *y*-axis
*F*_*Iy*_: The force acting at a point I in the direction of *y*-axis
*F*_*O*4*y*_: The force acting at point O_4_ in the direction of *y*-axis
*M*_*O4t*_: The bending moments at point O_4_
*M*_*It*_: The bending moments at point I
*M*_*Ht*_: The bending moments at point H
*δ*_3_: The displacement due to drift of beam 3
*θ*_*3*_: The angle variable created via the torque of beam 3
*K*_*O*4*x*_: The lateral bending stiffness by the force *F*_*O4y*_
*K*_3_: The I point's output stiffness
*K*_*O*4*t*_: The rotational stiffnesses of point O_4_
*K*_*It*_: The rotational stiffnesses of point I
*K*_*Ht*_: The rotational stiffnesses of point H
*λ*_3_: The amplification ratio of the amplifier of beam 3
*K*_*in*3_: The input stiffness of beam 3
*F*_*O*2*y*_: The force acting at point O_2_ in the direction of *y*-axis
*F*_*Cy*_: The force acting at point C in the direction of *y*-axis
*F*_*Jy*_: The force acting at point *J* in the direction of *y*-axis
*M*_*O*2*t*_: The bending moments at point O_2_
*M*_*Jt*_: The bending moments at point J
*M*_*Ct*_: The bending moments at point C
*δ*_2_: The displacement due to drift of beam 2
*θ*_*2*_: The variable angle created via the torque of beam 2
*K*_*O*2*x*_: The lateral bending stiffness by the force *F*_*O2y*_
*K*_*2*_: *E* point's output stiffness
*K*_*O*2*t*_: The rotational stiffnesses of point O_2_
*K*_*Ct*_: The rotational stiffnesses of point C
*K*_*Jt*_: The rotational stiffnesses of point J
*λ*_2_: The amplification ratio of the amplifier of beam 2
*K*_*in*2_: The input stiffness of beam 2
*F*_*O*1*y*_: The force acting at point O_1_ in the direction of *y*-axis
*F*_*Ay*_: The force acting at point A in the direction of *y*-axis
*F*_*By*_: The force acting at point B in the direction of *y*-axis
*M*_*O*1*t*_: The bending moments at point O_1_
*M*_*Bt*_: The bending moments at point B
*M*_*At*_: The bending moments at point A
*δ*_1_: The displacement due to drift of beam 1
*θ*_*1*_: The angle variable created via the torque of beam 1
*K*_*O*1*x*_: The lateral bending stiffness by the force *F*_*O1y*_
*K*_*1*_: The output stiffness combines A and B points
*K*_12_: The output stiffness combines beam 1 and beam 2
*K*_13_: The output stiffness combines beam 1 and beam 3
*K*_*y*12_: The intermediary stiffness between beam 1 and 2
*K*_*y*13_: The intermediary stiffness between beam 1 and 3
*K*_*O*1*t*_: The rotational stiffnesses of point O_1_
*K*_*Bt*_: The rotational stiffnesses of point B
*K*_*At*_: The rotational stiffnesses of point A
*λ*_1_: The ratio of the amplifier of beam 1
*k*_*in*1_: The input stiffness of beam 1
*K*_*θzMz*_^*l*7^: The stiffness created via the torque of the leaf hinge *l*_7_
*K*_*θyMy*_^*l*7^: The linear stiffness created via the axial force of the leaf hinge *l*_7_
*K*_*θzMz*_^*l*8^: The stiffness created via the torque of the leaf hinge *l*_8_
*K*_*θzMz*_^*l*9^: The stiffness created via the torque of the leaf hinge *l*_9_
*K*_*b*3_: The output stiffness combines *K*_*θzMz*_^*l*9^ and double *K*_*θzMz*_^*c*^
*ω*_1_: The rotational angle of the leaf hinge *l*_8_
*ω*_2_: The rotational angle of the leaf hinge *l*_7_
*ω*_3_: The rotational angle of the leaf hinge *l*_9_
*α*_1_: The rotational angle of flexible hinge of the amplifier of beam 4
*β*_1_: The rotational angle of the flexible hinge of the amplifier of beam 3
*γ*_1_: The rotational angle of the flexible hinge of the amplifier of beam 2
*ε*_1_: The rotational angle of the flexible hinge of the amplifier of beam 1
*d*_*out*_: The output displacement
*d*_*in*_: The input displacement
*E*_*p*_: The potential energy inside the flexure hinges
*K*_*out*_: The output stiffness of the micromanipulator
*K*_*N*_: The combine stiffness of the micromanipulator in one direction
*K*_*a*_: The combine stiffness of *K*_*θyMy*_^*l*7^
*σ*_*r*_^*Max*^: The maximal stress occurs when the angular displacement *θ*_max_
*σ*_*y*_: The yield strength of the manufactural material
*s*: The safety factor
*k*_*c*_: The concentration factor of a flexure circular notched hinge
*f*(*β*): The compliance factor of a flexure circular notched hinge
*β*: The dimensionless geometry factor of a flexure circular notched hinge
*δ*_*in*_^*Max*^: The maximal input displacement
*θ*_max_: The maximal deformation angle
*T*: The kinetic energy of the whole stage
*T*_*η*_1__: The kinetic energy of *y*-axis
*T*_*η*_2__: The kinetic energy of *x*-axis
*T*_*i*_: The kinetic energy of the element in the micromanipulator (*i* = *m*_1_ to *m*_b3_)
*η*_1_^.^: The coordinate used to describe the motions of *y*-axis
*η*_2_^.^: The coordinate used to describe the motions of *x*-axis.
